# Impulsivity Relates to Multi-Trial Choice Strategy in Probabilistic Reversal Learning

**DOI:** 10.3389/fpsyt.2022.800290

**Published:** 2022-03-14

**Authors:** Amy R. Zou, Daniela E. Muñoz Lopez, Sheri L. Johnson, Anne G. E. Collins

**Affiliations:** ^1^Department of Psychology, University of California, Berkeley, Berkeley, CA, United States; ^2^Institute of Human Development, University of California, Berkeley, Berkeley, CA, United States; ^3^Helen Wills Neuroscience Institute, University of California, Berkeley, Berkeley, CA, United States

**Keywords:** impulsivity, reversal learning, working memory, reinforcement learning, computational modeling

## Abstract

Impulsivity is defined as a trait-like tendency to engage in rash actions that are poorly thought out or expressed in an untimely manner. Previous research has found that impulsivity relates to deficits in decision making, in particular when it necessitates executive control or reward outcomes. Reinforcement learning (RL) relies on the ability to integrate reward or punishment outcomes to make good decisions, and has recently been shown to often recruit executive function; as such, it is unsurprising that impulsivity has been studied in the context of RL. However, how impulsivity relates to the mechanisms of RL remains unclear. We aimed to investigate the relationship between impulsivity and learning in a reward-driven learning task with probabilistic feedback and reversal known to recruit executive function. Based on prior literature in clinical populations, we predicted that higher impulsivity would be associated with poorer performance on the task, driven by more frequent switching following unrewarded outcomes. Our results did not support this prediction, but more advanced, trial-history dependent analyses revealed specific effects of impulsivity on switching behavior following consecutive unrewarded trials. Computational modeling captured group-level behavior, but not impulsivity results. Our results support previous findings highlighting the importance of sensitivity to negative outcomes in understanding how impulsivity relates to learning, but indicate that this may stem from more complex strategies than usually considered in computational models of learning. This should be an important target for future research.

## Introduction

Impulsivity, defined as the trait-like tendency to engage in rash, regrettable action (1), has been considered core to understanding problematic human behavior. Hundreds of studies have focused on the effects of this pernicious trait on psychopathology, aggression, and suicidality (2).

Not surprisingly, there has been a voluminous literature regarding the potential mechanisms involved in impulsivity. Self-control, the inverse of impulsivity, rests on the ability to over-ride reflexive responses to cues of potential reward and to emotional urges, and accordingly, theory has long focused on the idea that impulsivity might reflect some combination of poor constraint coupled with dysregulation in responses to reward cues and emotionality (3–5). This has led to a search for parallel cognitive processes involving executive control and reward processing as potential correlates of impulsivity.

Regarding executive control, many studies have considered response inhibition and working memory capacity as potential correlates of impulsivity. Response inhibition, defined as the ability to inhibit motoric responses to prepotent stimuli, has been theorized to be of import given that emotions and cues of reward might be considered prepotent stimuli, such that control is needed to override reflexive, immediate responses. Findings regarding the link between impulsivity and response inhibition, though, have been mixed, meta-analytic *r* < 0.10 (6), with one meta-analysis observing moderate effects only within clinical samples (7).

Working memory is the ability to actively and effortfully maintain a small amount of information (the working memory *capacity*) for a short amount of time, and to shield that information from interference from competing information; it plays a critical role in self-regulation (8, 9). Although some research has suggested that Barratt Impulsivity scores (BIS-11) (10) are related to lower working memory capacity (11), most authors have observed null effects of the BIS-11 scale with working memory tasks, including the digit span task (12, 13), the N-back task (14–16), the CANTAB test of spatial working memory (11, 17), the O-Span task (18), or a composite of well-validated working memory tasks (19). In parallel, findings have been mixed on whether working memory training lowers BIS-11 scores (20, 21).

Some findings suggest that more precision in the measurement of impulsivity may inform this work. Considerable research highlights that self-rated dimensions of impulsivity are only modestly correlated (22–24) and relate differentially to psychopathology and behavioral outcomes (2). One key form of impulsivity not captured by the BIS-11 is Negative Urgency, a subscale of the UPPS scale that captures tendencies to respond with rash speech and behavior in the face of negative emotion (25). Negative Urgency is statistically separable from other self-rated forms of impulsivity, such as lack of Perseverance, lack of Premeditation, and Sensation-seeking, and it appears much more robustly tied to psychopathology and behavioral problems than do those forms of impulsivity (2). A separate subscale of the UPPS, Positive Urgency, covering tendencies to respond rashly to positive emotion states, was identified more recently (23), and also correlates robustly correlations with psychopathology (2, 26, 27). Large-scale factor analytic and network models suggest that Positive and Negative Urgency are closely related and form a higher order factor (22, 28).

When using the UPPS scale, several researchers have found that lower working memory performance is negatively related to specific forms of impulsivity. That is, researchers have observed negative correlations of the Negative Urgency scale with working memory as assessed using the OSPAN (29), the Digit Span task (30), and the Operation Word Span task (31). Across two studies, (lack of) Perseverance has been found to be related to significantly lower working memory performance, using the Digit Span Task (30) and the Letter-number Sequence task (32). Further investigation, though, is warranted as null effects have been reported for Urgency, (lack of), Perseverance and the other UPPS scales with working memory indices, including the Digit Span task (33), the Spatial Working Memory task (34), the Letter-number Sequence task (35), and a composite of three working memory tasks (36).

Beyond a focus on working memory, considerable research has examined how self-rated impulsivity relates to dysregulated or excessive pursuit of rewards (37, 38). Not all facets of reward processing, though, appear to be tied to all facets of impulsivity. Although a preference for immediate over long-term rewards has often been framed as a behavioral index of impulsivity, findings relating delay discounting tasks to BIS scores have been mixed, with positive (39) and null findings (13).

Reinforcement learning (RL) is a cognitive process that uses rewards and punishments to learn which actions are beneficial over time (40). Researchers have observed ties between RL and self-rated impulsivity. For example, researchers have shown that impulsivity is tied to gambling tasks in which a person must learn about potential rewards, with negative correlations of the I7 with the Iowa Gambling task (37), and of premeditation scores (UPPS-P) on a different gambling task (41). In another study, Negative Urgency and (lack of) Perseverance, but not Persistence or Sensation-seeking, significantly related to diminished reward learning on the IGT (42). To date, these effects appear tied to learning, in that BIS-11 scores did not correlate significantly with performance on a card task that provided labels for decks with short vs. long term rewards (14). Accordingly, we focus here on reward-seeking tasks that involve learning.

Among reward-seeking tasks that involve learning, reversal learning tasks, and in particular probabilistic reversal learning tasks, offer an ideal test-bed for investigating the role of impulsivity in decision making. Theories of impulsivity have focused on the importance of the interaction of executive function in combination with reward-seeking [cf. (4, 31)]. Probabilistic reversal learning tasks necessitate executive function, for example in the form of response inhibition: when a negative outcome occurs, participants may need to actively refrain from switching as it may signal noise rather than a reversal.

Consistent with theory, multiple studies have shown that impulsivity is tied to performance on versions of reversal tasks, most commonly in samples oversampled for clinical conditions related to impulsivity, such as gambling or alcohol addiction. Those studies have shown that performance is lower on reversal learning tasks among those with high impulsivity, as measured using the I7 (37) or BIS-11 (11, 43–45). In one study, Negative Urgency, but not other UPPS scales, was significantly correlated with more perseverative errors on a response reversal task (46), again highlighting the import of specificity in definitions of impulsivity.

Much remains unknown, however, regarding links of impulsivity dimensions with performance in probabilistic reversal tasks. First, researchers have rarely considered these links within healthy populations. Second, previous studies that showed links of impulsivity with reversal performance often provided deterministic switch information despite probabilistic outcomes across a range of values (11, 37, 43, 47). Furthermore, these studies used outcomes of variable amounts, such that participants needed to consider not only uncertainty but also expected outcomes, which could account for some of the findings. Other studies, such as Clatworthy et al. (44), did test probabilistic reversal with binary outcome, so that the switch could not be deterministically observed and had to be inferred, and did not observe a main effect of impulsivity, but an interaction with a dopaminergic drug, such that more impulsive participants' reversal performance benefited more from the drug; however, there was a single reversal in this task, limiting statistical power. In a probabilistic learning task, Sali et al. (48) observed that more impulsive participants were more likely to switch after a negative outcome and less likely to stay after a positive outcome, showing more sensitivity to reward; however, this was not in a switching context, and independent of whether the participants had experienced switches in task contingencies previously or not.

Thus, the role of impulsivity in probabilistic reversal learning remains unclear. Here, we use a simple probabilistic reversal learning task to probe the role of impulsivity in flexible decision making under uncertainty in dynamically changing environments. Our design allows us to test the impact of uncertain feedback on choice stability (or switching behavior) when participants need to infer if a negative outcome signals change or noise, in the absence of confounds about outcome amounts, as often used in gambling tasks. We predicted that more impulsive participants would be more likely to switch after a negative outcome [as observed in (48)], and as such, would have overall worse performance. We also tested whether there was a link between impulsivity and another form of executive function (working memory), in another task (not discussed here) enabling us to identify working memory. Our results do not support our predictions in probabilistic reversal learning, but reveal strong, subtler effects of impulsivity on dynamic choice behavior in probabilistic reversal learning, along with no effect on working memory contributions to learning.

## Methods

### Participants

This research was approved by the University of California, Berkeley Institutional Review Board. All participants provided written informed consent. Participants were students who received partial credit in their psychology classes at the University of California, Berkeley. Eligible participants were native or fluent English speakers between the ages of 18 and 40 with no self-significant history of brain injury, mental/psychiatric illness such as Parkinson's disease, OCD, schizophrenia, depression, ADD/ADHD, or alcohol or drug abuse per self-report. Our initial sample size was 102. After excluding those who failed to meet inclusion criteria, did not complete the entire task, or performed below criterion, our sample size was 86. A subset of participants did not perform the second block of the task; for analyses including both blocks jointly, we thus limited analyses to participants who completed both blocks, for a final sample size of 70 (age = 20.7 ± 2.19 years, 75.7% female, 24.3% male).

### Probabilistic Switching Task Design

Participants completed two independent learning tasks in a single 1-h session: the RLWM task with a learning and testing phase (49, 50), and the probabilistic reversal learning task (one or two blocks, depending on time). Participants first completed the RLWM learning phase (a deterministic stimulus-action association learning task); then one block of the probabilistic reversal learning task, then the testing phase of RLWM, then, if time remained, a second block of the probabilistic reversal learning task. Here, we focus on the probabilistic reversal learning task (see Supplementary Information for methods and results on the RLWM task).

Participants completed the probabilistic switching task online and remotely from their own computer (51, 59). The task was written using the Javascript library jsPsych (52), and the webpage was hosted by Jetstream (53, 54).

**Figure 1:**
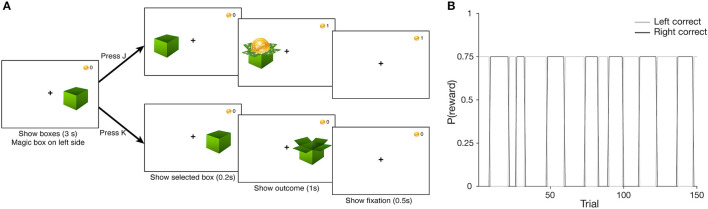
**(A)** Probabilistic Switching task design. Participants saw two boxes at the start of the trial, and had 3 s to choose a box *via* key press (“J” to select the left, “K” for the right). After a 0.2 s delay, where only the selected box was shown, the outcome was shown for 0.5 s—a coin if the participant was rewarded, and an empty box otherwise; points total was updated in the top right corner of the. Finally, a screen with only the fixation cross was shown for 0.5 s to signal the start of the upcoming trial. **(B)** Task block structure. The correct box identity reverses unpredictably over a block. Correct box selection leads to a coin with a reward probability *p*(*rew*) = 0.75, while selecting the incorrect box never leads to a coin [*p*(*rew*) = 0].

In the task, participants were presented with two boxes on each side of the screen. We instructed them to find the “magical” box that would reward them most of the time, and that the “magical” box would occasionally switch sides throughout the task (see Supplementary Information for specific instructions). Participants used the “J” and “K” keys to select the left and right box, respectively. A given trial proceeded as follows. First, participants saw both boxes and had 3 s to make a selection. Once a choice was made, the selected box was shown for 0.2 s, followed by the outcome (box containing a coin for reward, or an empty box for no reward) for 1 s. Finally, a fixation cross was shown for 0.5 s to signal the start of the next trial (Figure 1A). A block consisted of 150 trials (average of 6.11 min), and participants completed a second block as time permitted. The blocks of the probabilistic switching task were weaved between the phases of another task [RLWM from (49)], which will not be discussed in this paper; participants who completed both blocks did so with a few minutes of delay in between.

The correct box was always on the left side at the start of a block, with a reward probability of 75%. The reward probability of the incorrect box was always 0%. A pre-randomized sequence of reward outcomes on correctly answered trials was loaded at the start of the task (i.e., reward outcomes were not sampled online, to ensure a better match to the true probabilities). Upon accumulating a certain number of rewards on one side, the correct box would switch to the other side immediately following a correct, rewarded choice. The number of required rewarded trials to switch was sampled randomly from a range of 5–15 uniformly, and this sequence of switch criteria was loaded at the start of the task (i.e., sampled offline for the same reason listed above, Figure 1B). We created four versions of each sequence and randomly assigned them to participants at the start of the task. Participants who completed two blocks experienced a different version on each block.

### Measure of Impulsivity

To measure impulsivity, we used the Short Version of the UPPS-P Impulsive Behavior scale, measuring Urgency, Premeditation (lack of), Perseverance (lack of), Sensation Seeking, and Positive Urgency (S-UPPS-P) (55). This 20-item self-report questionnaire has been found to replicate the internally consistency (0.74–0.88 across subscales) and inter-scale correlations of the original full 59-item UPPS-P scale Positive Urgency (S-UPPS-P) (55). Additionally, the S-UPPS-P subscales strongly correlate with the full UPPS-P subscales: negative urgency (*r* = 0.69), positive urgency (*r* = 0.83), lack of perseverance (*r* = 0.63), lack of premeditation (*r* = 0.71), and sensation seeking (*r* = 0.64) (55). Items, e.g., “Once I get going on something I hate to stop” and “I tend to lose control when I am in a great mood,” are rated on a scale of 1 (strongly agree) to 4 (strongly disagree), with 12 reverse scored items, such that higher scores reflected more impulsivity. Total impulsivity scores ranged from 26 to 63 (possible range 20–80) and were normally distributed (W = 0.98, *p* = 0.38). The total scale and subscales showed good internal consistency reliability (total α = 0.83, negative urgency α = 0.77, premeditation α = 0.83, perseverance α = 0.75, sensation seeking α = 0.78, and positive urgency α = 0.80).

### Behavioral Analysis

Correlations between impulsivity and behavioral measures were conducted in MATLAB (56). Generalized linear mixed-effects models were run in R Studio (57) using the lme4 package (58).

We used a logistic mixed effects model to predict trial action (dependent variable) from positive and negative outcomes up to three trials in the past (six independent variables), accounting for random effects across participants. The formula is as follows:


$$\begin{array}{l} action\;\sim \;pos\text{\_}1\;+\;neg\text{\_}1\;+\;pos\text{\_}2\;+\;neg\text{\_}2\;+\;pos\text{\_}3\;\\ +\;neg\text{\_}3\;+\left(1\;|\;id\right) \end{array}$$

Trial actions were coded as 0 for selecting the left box, and 1 for selecting the right. For a delay of *i, pos_i* had a value of −1 if the left box was rewarded on trial *t–i*, 1 if the right box was rewarded on *t–i*, and 0 if a positive outcome did not occur on that trial. Similarly, *neg_i* would have a value of −1 if the left box was unrewarded on *t-i*, 1 if the right box was unrewarded on *t–i*, and 0 if a negative outcome did not occur on that trial.

To comprehensively account for impulsivity and its interactions with effects of past outcomes, we constructed the following model using a *z*-scored total impulsivity score:


$$\begin{array}{l} action\;\sim \;impulsivity\;+\;impulsivity\;(pos\_1\;+\;neg\_1\;\\ \;+\;pos\_2\;+\;neg\_2\;+\;pos\_3\;+\;neg\_3)\;+\;\left(1\;|\;id\right) \end{array}$$

This model accounts for a main effect of impulsivity, and six interactions for each past outcome predictor. We chose a maximum value of *i* = 3 based on previous research showing that most variance was accounted for with the last three trials (Eckstein et al., submitted); however, we obtained similar results with a higher number of past trials taken into consideration.

### Statistical Analysis

For correlation analyses, we used Pearson correlation unless the variables were not normally distributed according to the Shapiro–Wilk test at α = 0.05, in which case we used Spearman correlation. We did not correct for multiple comparisons for our *a priori* core hypotheses. For exploratory analysis of *a posteriori* behavioral markers, we report where results survive multiple comparison. For exploratory analysis of correlations with subscales, we report results uncorrected for multiple comparisons.

### Computational Modeling

We used computational modeling to analyze underlying cognitive processes behind the task behavior in a way that qualitative, model-free analyses cannot. To model the behavioral data of this task, we selected the reinforcement learning (RL) framework and Bayesian inference (BI) framework, both of which are grounded in behavior-based theory and generalizable to various behavioral tasks.

#### Reinforcement Learning

Reinforcement learning is the process through which an agent learns about the values of actions and stimuli from their outcomes. Under this framework, we assume that the agent learns about choices in an incremental fashion *via* reward-prediction errors and uses a policy that maximizes reward by choosing the more valuable action.

We used Q-learning to model the value-learning process in our task. The expected value of an action *Q*(*a*) is updated by the difference between the previous reward outcome *r* and prior expectations from the previous time point *t*. This difference is called the reward prediction error and is scaled by a learning rate α between 0 and 1. A learning rate closer to 0 makes for a more gradual update, while one closer to 1 more heavily weighs the immediate previous outcome. Specifically, for each action *a* (pick left vs. pick right), the update is:


$$\begin{array}{l} Q_{t+1}\left(a\right)=Q_{t}\left(a\right)+\alpha \times \left[r_{t}-Q_{t}\left(a\right)\right] \end{array}$$

We calculated probabilities of action selection using softmax and the inverse temperature parameter β as follows:


$$P_{t}(a)=\frac{\exp(\beta Q_{t}(a))}{\exp(\beta Q_{t}(a)+Q_{t}(a_{un})))}$$

To capture asymmetrical learning across positive and negative outcomes, we parameterized feedback-dependent learning through separate learning rates α for positive outcomes and α_−_ for negative outcomes:


$$\begin{array}{l} \;Q_{t+1}\left(a\right)=Q_{t}\left(a\right)+\alpha \left[r_{t}-Q_{t}\left(a\right)\right],\;r_{t}=1\\ Q_{t+1}\left(a\right)=Q_{t}\left(a\right)+\alpha_{-}\left[r_{t}-Q_{t}\left(a\right)\right],\;r_{t}=0 \end{array}$$

We also considered mechanisms that enable faster learning, in particular counterfactual updating. Specifically, we simultaneously updated the value of the unchosen action *a*_*un*_ with the opposite counterfactual outcome. In our winning model, replicating Eckstein et al. (submitted), we did not use a separate learning rate for counterfactual learning. Accordingly, counterfactual updating for *a*_*un*_ was:


$$\begin{array}{l} \;Q_{t+1}(a_{un})=Q_{t}(a_{un})+\alpha [(1-r_{t})-Q_{t}(a_{un})],\;r_{t}=1\\ Q_{t+1}(a_{un})=Q_{t}(a_{un})+\alpha_{-}[(1-r_{t})-Q_{t}(a_{un})],\;r_{t}=0 \end{array}$$

To capture stickiness in action selection, a heuristic where the agent repeats a selected action, we included a sticky choice parameter *st* that boosts the value of the previously selected action before action selection on the current trial. Thus, the weights passed to the softmax policy were *W*(*a*) = *Q*(*a*)+*st* if *a* = *a*__*t*_ − 1_, and *W*(*a*) = *Q*(*a*) otherwise. Values of *st* lie between −1 and 1, with positive values capturing tendencies to stay with a choice, and negative values capturing tendencies to switch to the other choice.

We also fit the simpler models in the RL nested model family (i.e., simplest being one feedback-independent α and one β) for the sake of model comparison, however, we will not discuss them here. Model comparison confirmed findings in a recent study with the same task that this model best accounted for the data (Eckstein et al., submitted); see model comparison plots in Supplementary Figure S4B. Thus, our RL model consists of four parameters.

#### Bayesian Inference

Behavior in probabilistic reversal tasks has often been described as an inference problem, rather than a value learning problem (59, 60). Under this framework, the agent infers latent states in its environment from what they can observe and uses this to update their beliefs about the latent states to make better predictions. In the context of this task, the latent state is *C*_*t*_ (*C*_*t*_ = left or right), the identity of the “correct” box at time *t*. The agent tracks its belief that the correct box is left or right *p*(*C*_*t*_=*left*) at each trial. To do so, it updates its belief according to Bayes' rule, based on its prior belief and the likelihood of the observed evidence at each trial (the outcome *r*_*t*_ of a given choice *a*_*t*_). The prior belief reflect the agent's model of the task, including knowledge that *C*_*t*_ might change at each trial. Using this model, the agent calculates the posterior probability over the latent state *via* Bayesian updating. Specifically:


$$\begin{array}{l} P\left(C_{t}=i|r_{t},a_{t},H_{t-1}\right)=\frac{P\left(r_{t}|C_{t}=i,a_{t}\right)P\left(C_{t}=i|H_{t-1}\right)}{\sum_{j}P\left(r_{t}|C_{t}=j,a_{t}\right)P\left(C_{t}=j|H_{t-1}\right)} \end{array}$$

Where *H*__*t*−_1_ is the reward and choice history up to trial *t–*1, and *i, j* are in [left, right]. The likelihood *P*(*r*_*t*_|*C*_*t*_ = *i, a*_*t*_) is defined according to whether choice matched the latent state:


$$\begin{array}{l} p\left(r_{t}=1|a_{t}=i,\;C_{t}=i\right)=p_{reward}\\ \;p\left(r_{t-1}=1|a_{t}\neq i,\;C_{t}=i\right)=\varepsilon \end{array}$$

Where *p*_*reward*_ a model parameter indicating the probability that the correct box will be rewarded, and ε is fixed to a small value 0.0001 to represent the participants' knowledge that the incorrect box never gives reward outcomes, but not to 0 to avoid model degeneracy.

Before a choice, this posterior belief for the last trial's correct box is updated to a prior belief for the upcoming trial according to model parameter *p*_*switch*_, the probability that a switch may have occurred on this trial.


$$\begin{array}{l} p\left(C_{t+1}=i\;|\;H_{t}\right)=\left(1-p_{switch}\right)\;p\left(C_{t}=i\;|\;H_{t}\right)\\ +p_{switch}\left(1-p\left(C_{t}=i\;|\;H_{t}\right)\right) \end{array}$$

Choice policy for the BI model is identical to the RL model, with beliefs replacing learning value. It also includes sticky choice behavior:


$$\begin{array}{l} W_{i}\left(t+1\right)=p\left(C_{t+1}=i\;|\;H_{t}\right)\;+\;st\left(i=a_{t}\right)\\ P\left(a_{t+1}=i\right)=\frac{\exp\left(\beta W_{i}\left(t+1\right)\right)}{\sum_{j}\exp\left(\beta W_{j}\left(t+1\right)\right)} \end{array}$$

Variants of this model with fixed *p*_*reward*_ and *p*_*switch*_ were explored in Eckstein et al. (59) and could not account for behavior well.

#### Model Fitting

We used hierarchical Bayesian modeling to fit models to the data. Under a hierarchical framework, we assume that subject-level parameters are drawn from a group-level distribution. A hierarchical approach helps improve accuracy of subject-level parameter values (61). In particular, sampling subject-level parameters from a group-level distribution regularizes the process and helps to avoid estimates at extreme parameter boundaries (e.g., a learning rate of 1).

For the RL model (Supplementary Figure S4), individual participants' α and α_−_ parameters were sampled from group-level beta distributions, and their hyperparameters were sampled from gamma(1,1); β parameters were sampled from a gamma distribution, and its hyperparameters were sampled from gamma(1,1); individual *st* parameters were sampled from a group-level normal distribution, with mean μ sampled from N(0,10) and standard deviation σ from half-Normal(0, 10).

For the BI model, individual parameters *p*_*reward*_ and *p*_*switch*_ were sampled from group-level beta distributions, and their hyperparameters were sampled from gamma(1,1). Individual parameters β and *st* and their hyperparameters were sampled in the same way as their RL model counterparts (see Figure S4A for graphical model of parameters and priors).

We used Markov Chain Monte Carlo (MCMC) sampling to estimate parameter values from the posterior distribution, using the Stan software (62) interacting with MATLAB *via* matstanlib (63) and MatlabStan (64). To fit each model, we ran 4 chains, with 1,000 warm-up trials and 5,000 iterations for each chain. We verified that there were no divergences, that all Rhat values were under 1.01, and effective sample size > 40, ensuring that the posterior distribution over each parameter was well-approximated (65). We also verified *via* generate and recover procedures that the model parameters were recoverable (66). For model comparison, we calculated the Watanabe–Akaike Information Criterion [WAIC from (67)] and compared scores across all models in the RL family to account for overparameterization. WAIC averages over the posterior distribution instead of conditioning on a point estimate, which is what AIC and BIC condition on, and is thus more appropriate for our hierarchical Bayesian modeling approach (68). Indeed, the RL model using all 4 parameters described above fit best. Since the purpose of including the BI model was to replicate previous findings that a BI model could capture behavior as successfully as RL could (59), we also compared WAIC scores of the best-fitting RL model and the BI model, which yielded a much smaller difference (see Supplementary Materials for details).

To validate our models, we simulated behavioral data for each participant using the point-estimates of their respective parameters (expected value), then analyzed the resulting data in the same way as the empirical data. We verified that the winning models were able to capture important qualitative patterns in the data.

## Results

### Group-Level Switching Behavior

**Figure 2:**
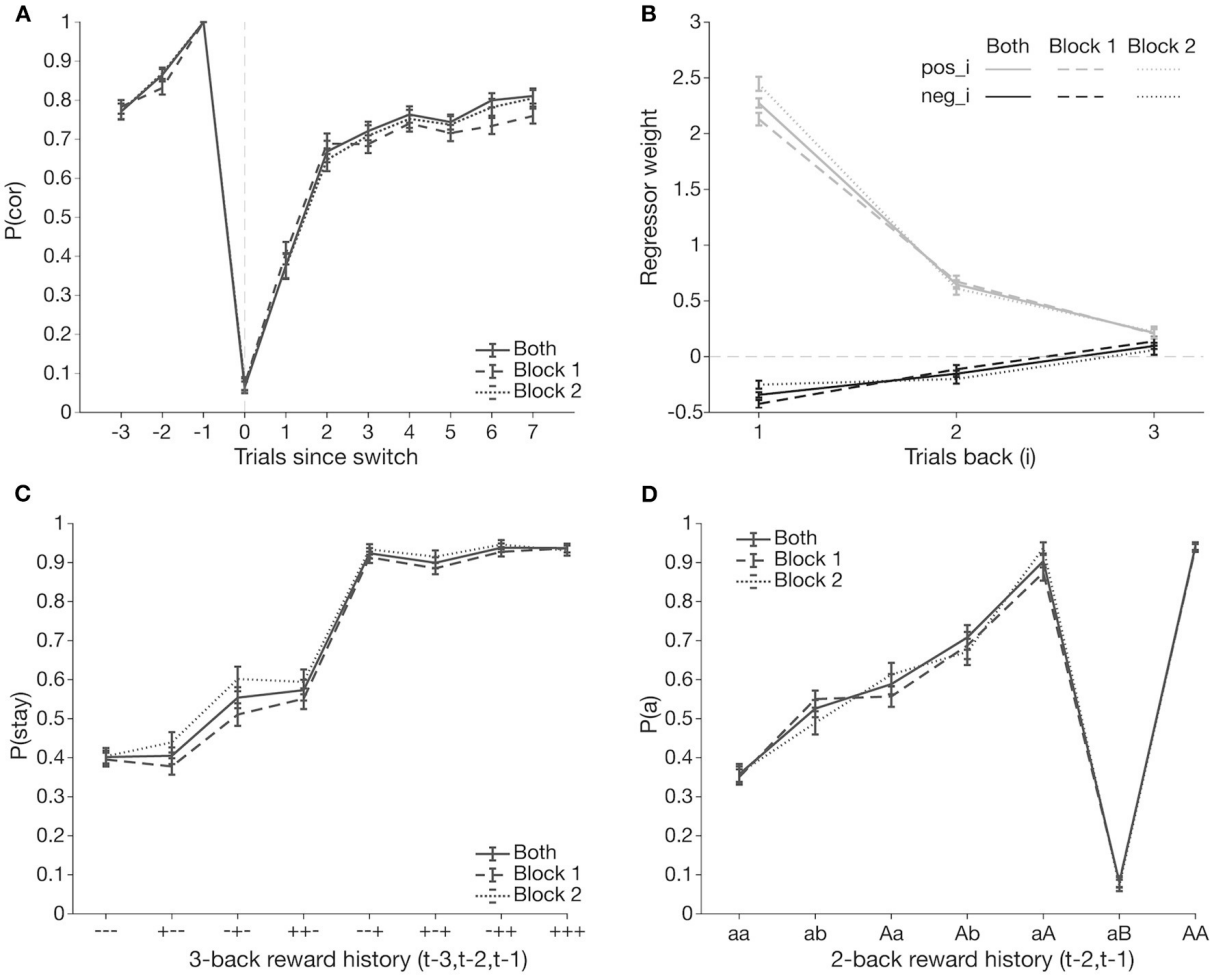
Group level switching behavior. Joint block behavior (solid line) is comparable to behavior from only block 1 (dashed line) and only block 2 (dotted line). Data points are averaged over all participants, bars represent standard error of the mean. **(A)** Probability of selecting the correct box [*p*(*cor*)] surrounding a switch trial (*t* = 0). **(B)** Comparison of regressor weights from GLME predicting an action based on reward outcomes from up to three trials past (*t*−3). Past positive outcomes (*pos_i*, light) are more likely to induce selecting the same action; a negative outcome (*neg_i*, dark) at *t*−1 has the greatest effect to induce a switch. **(C)** Probability of repeating the previous trial's choice [*p*(*stay*|- - -)] as a function of three trial-back reward histories [*t*−3, *t*−2, and *t*−1]. **(D)** Probability of selecting an action *a* at trial *t* given that was selected two trials ago (*t*−2), across all possible action and reward outcomes on *t*−1 [*t*−2, *t*−1]. “b” indicates selection of the other action; lower/upper case indicates whether the action was rewarded/unrewarded.

Behavior across both blocks was comparable (Figure 2). Thus, for further analyses, to increase within-participant power, we focused on 70 participants who completed two blocks of the task and analyzed behavior from both blocks jointly (Figure 2). Participants exhibited switching behavior consistent with group-level findings from previous versions of the task: they adjusted their action selection within 2–3 trials following a switch trial and achieved asymptotic performance around 75% accuracy after successfully switching (Figure 2A).

To investigate the effect of previous outcomes on action selection, we analyzed *p*(*stay*|- - -), the probability that the selected action on trial *t* is the same action on trial *t–*1, based on outcomes from the past two trials (coded as a sequence of 2 characters; “+” = reward, “–” = no reward, first symbol = outcome at *t–*2, and second symbol = outcome at *t*−1). Participants almost always stayed with an action, if it was previously rewarded, and tended to switch after two negative outcomes. However, after a single negative outcome, the choice policy was much more variable, with equal probability of staying and switching, as observed in previous research (Supplementary Figure S1).

To quantitatively investigate how past outcomes affected action selection, we used a mixed effects logistic regression analysis predicting action selection from the past three outcomes (see section **Methods** for details). All predictors had significant main effects, with stronger effects of positive than negative outcomes (Figure 2D; Table 1). Effects of past trials decreased with distance to the current trial. These results suggest that past positive outcomes, especially at *t–*1, drive repeating an action, while past negative outcomes drive switching behavior.

**Table 1:** Statistical output from the GLME model with impulsivity interactions fit over both blocks jointly across participants (*n* = 70).

	**Action**			
**Predictors**	**Estimate**	**SE**	***z***	***p***
(Intercept)	−0.10	0.03	−3.08	0.002
imp_score_z	0.00	0.03	0.09	0.931
pos_1	2.27	0.04	54.44	<0.001
neg_1	−0.35	0.02	−14.16	<0.001
pos_2	0.65	0.04	17.19	<0.001
neg_2	−0.16	0.03	−5.54	<0.001
pos_3	0.21	0.03	6.36	<0.001
neg_3	0.10	0.03	3.42	0.001
imp_score_z * pos_1	0.02	0.04	0.44	0.660
imp_score_z * neg_1	−0.10	0.03	−3.90	<0.001
imp_score_z * pos_2	0.04	0.04	1.03	0.304
imp_score_z * neg_2	0.00	0.03	0.02	0.985
imp_score_z * pos_3	0.02	0.04	0.70	0.486
imp_score_z * neg_3	0.02	0.03	0.82	0.414
	**Random effects**			
σ^2^		3.29		
τ_00__id_		0.05		
ICC		0.02		
N _id_		70		
Observations		20,937		
Marginal *R*^2^/Conditional *R*^2^		0.557/0.564		

*There was a significant main effect of impulsivity on neg_1 (negative outcome at t−1)*.

### No Effect of Impulsivity on *a Priori* Measures

**Figure 3:**
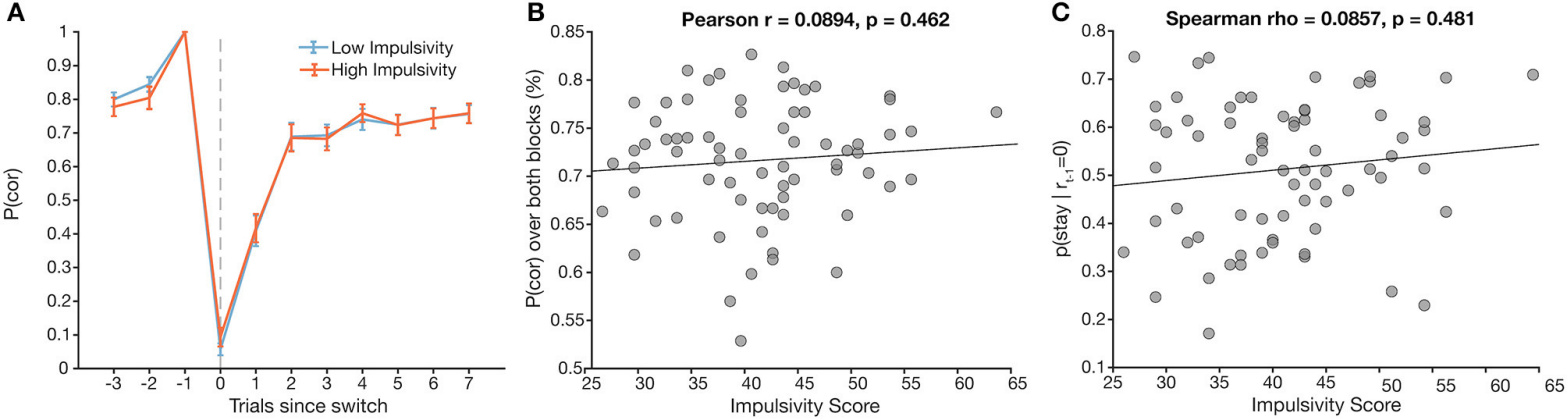
Total impulsivity score does not relate to *a priori* behavioral measures. **(A)** Task performance [*p*(*cor*)] around a switch trial plotted across low (blue) and high (orange) impulsivity groups (assigned *via* median split). **(B)** Total impulsivity score vs. overall task performance (*r* = 0.09, *p* = 0.46). **(C)** Total impulsivity score vs. probability of staying after an unrewarded trial (ρ = 0.09, *p* = 0.47).

To test our *a priori* hypotheses, we correlated total impulsivity scores with two key behavioral measures of interest: overall task performance (number correct/total trials answered in a block) and the probability of switching given that the previous trial was unrewarded. Correlations yielded no significant correlation for either behavioral measure (Figure 3B, Pearson *r* = 0.089, *p* = 0.46; Figure 3C, Spearman ρ = 0.086, *p* = 0.50).

**Figure 4:**
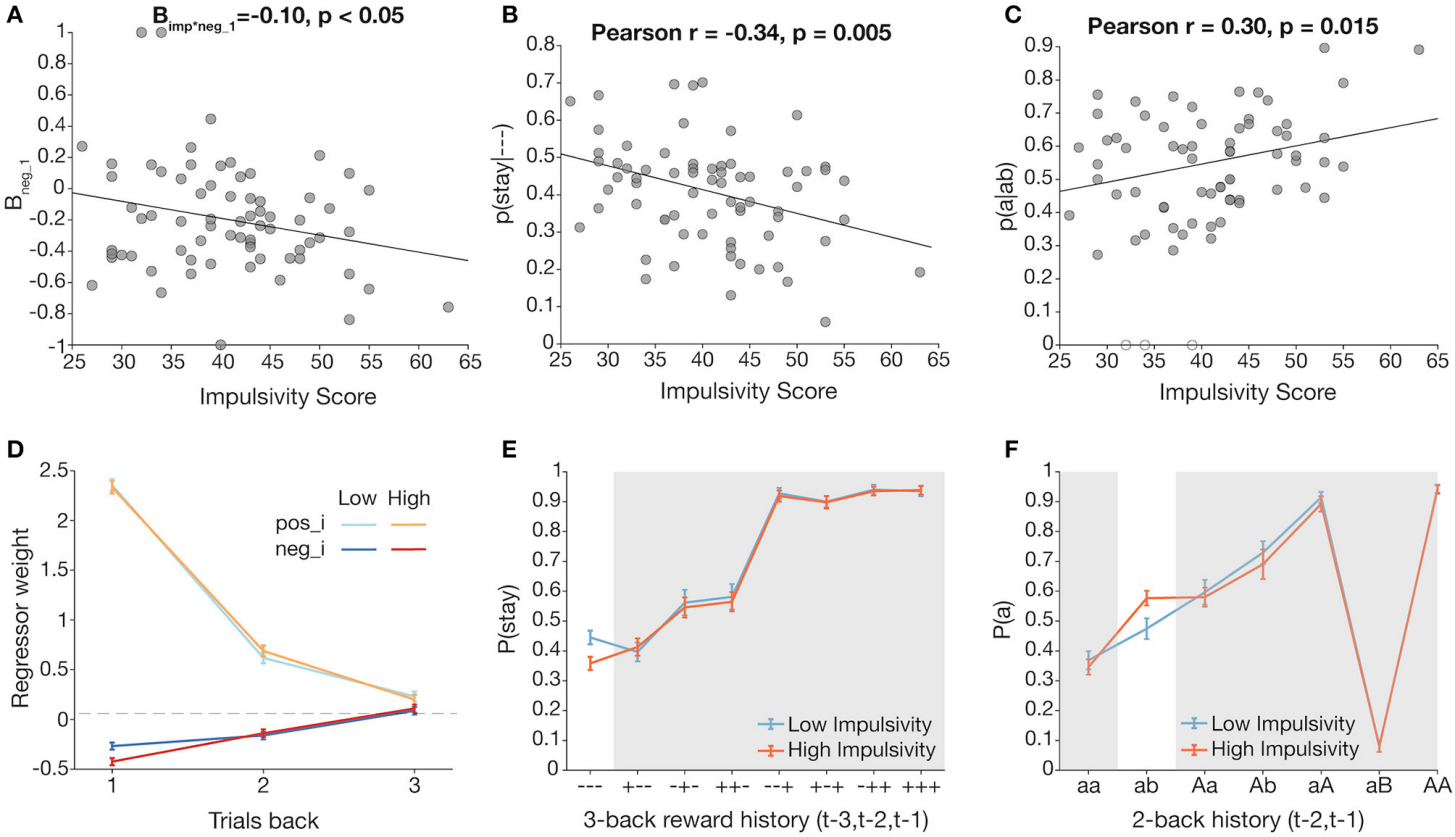
Trial history-dependent analyses reveal effects of impulsivity on staying behavior following unrewarded trials. Pearson correlations were conducted for normally distributed measures. **(A)** Total impulsivity score vs. normalized neg_1 (negative outcome at *t*−1) regressor weights, obtained by fitting a multinomial logistic regression over data by participant. **(B)** Total impulsivity score is significantly related to *p*(*stay*|- - -), the probability of staying following three unrewarded trials. **(C)** Total impulsivity score is significantly related to *p*(*a|ab*), the probability of returning to a previously unrewarded action following an unrewarded switch. Unfilled data points represent outlier participants. **(D–F)** Visualizations of impulsivity effects *via* median split (low impulsivity = blue, high impulsivity = orange). For **(D,E)**, highlighted columns of plots correspond to the scatterplot directly above. **(D)** Comparison of *neg_i* (dark) and *pos_i* (light) regressor weights (1 ≤ *I* ≤ 3) with median split for visualization. A negative outcome on *t*−1 has a greater effect on switching for the high impulsivity group. **(E)** Three-back analysis with median split for visualization. **(F)** aB analysis with median split for visualization.

We also constructed a logistic mixed effects model with normalized impulsivity score as a predictor that also interacted with each of the outcome predictors (Table 1). There was a significant effect of impulsivity on *neg_1*, such that the effect of negative feedback was stronger in more impulsive participants, in line with our main hypothesis, but conflicting with the previous, simpler analysis (Figure 4A). We further explore this finding in the next section.

In exploratory analyses, we tested whether our core behavioral measures were correlated with impulsivity subscale scores. The correlation between lack of Perseverance scores and task performance was significant (Spearman ρ = 0.25, *p* = 0.035), but did not survive multiple comparisons correction at *p* < 0.01; remaining correlations were not significant (all *p*s > 0.05; see Supplementary Figure S2 and Supplementary Tables S1, S2 for subscale score distributions and correlations).

### Exploratory Trial History-Dependent Analyses Reveal Effects of Impulsivity

Our analyses thus far did not consider longer trial history or potential interactions between past trial outcomes. Thus, we performed exploratory analyses to investigate their effect. We first conducted a 3-trial back analysis, computing staying behavior as a function of sequences of outcomes over the past three trials (Figure 2C). Consistent with our logistic mixed effects model results, staying behavior was highest after a reward on trial *t–*1, and driven by a reward on *t–*2 when *t–*1 was unrewarded.

We conducted correlations between total impulsivity score and conditional *p*(*stay*|- - -) from the new 3-trial back analysis (51, 59). There was a significant effect of impulsivity on *p*(*stay*|- - -) in the—condition, such that more impulsive participants were more likely to switch after three consecutive unrewarded outcomes (Figure 4B; Pearson *r* = −0.34, *p* = 0.0046; this survived a Bonferroni correction for eight analyses at *p* = 0.05). To better visualize this effect, we used a median split to form low and high impulsivity groups, and plotted 3-trial back behavior for each group (Figure 4E). Indeed, *p*(*stay|- - -*) was lower for the high impulsivity group, confirming that more impulsive participants were more likely to switch after being unrewarded three times in a row.

A caveat of the 3-trial back analysis is that it overlooks what actions were specifically selected in those past trials. Consider the cases where a participant is unrewarded for (A) switching from an unrewarded action, vs. (B) choosing to stay with an unrewarded action. The reward history is the same in both cases (unrewarded twice in a row), but decision contexts are different in that A may be a direct employment of the win-stay lose-switch strategy, while B could indicate more caution or sticky behavior.

To account for interactions between past trial outcomes and the actions resulting in those outcomes (i.e., whether switching in previous trials affects switching on trial *t*), we further subdivided trial history into categories that distinguish conditions not only by outcome, but also by action (69). Because we only have up to 300 trials per participant, we limited the scope of this action-based analysis, henceforth referred to as the aB analysis, to 2-trial back history (3-trial back would lead to bins with too few trials each). We calculated *p*(*a*), the probability that the selected action on trial *t* is the same as that on trial *t–*2, conditioned on the outcomes of *t*–1 and *t–*2 and whether a switch occurred at *t–*1, over all possible 2-trial history permutations (“aa,” “ab,” “aA,” “aB,” “Aa,” “Ab,” “AA,” and “AB”) (Figure 2D). Letter case indicates reward outcome, and “b” indicates a switch on *t–*1. Indeed, this analysis visualizes well the interaction between outcome and choice at *t–*1; for example, the considerable difference between *p*(*a|aA*) and *p*(*a|aB*) is consistent with the win-stay lose-switch strategy but would not appear in the 3-trial back analysis.

To clarify whether intermediate switching contributed to the impulsivity-driven differences seen in *p*(*stay*|- - -), we focused on the cases *p*(*a|aa*) and *p*(*a|ab*) (i.e., being unrewarded again for staying with an unrewarded action vs. being unrewarded for switching from an unrewarded action). Correlations of total impulsivity score and each measure revealed a significant correlation of impulsivity and *p*(*a|ab*) (Figure 4C; Pearson *r* = 0.30, *p* = 0.015, survives Bonferroni correction for two comparisons at *p* < 0.05), but not of impulsivity and *p*(*a|aa*) (see Supplementary Materials). This analysis excluded three participants who had outlier values of *p*(*a|ab*) = 0, i.e., they never returned to action *a* after a 2-back history of “ab”; inclusion of those three participants strengthened the statistical result. Indeed, applying the previous median split for the aB analysis showed a higher *p*(*a*) for the higher impulsivity group (Figure 4F), suggesting that following an unrewarded switch, more impulsive participants are more likely to reselect the action from trial *t–*2, effectively committing a double switch, rather than sticking with their previous decision to explore another side.

We fit our logistic mixed effects model across each subgroup to better visualize the interaction of *neg_1* and impulsivity score (Figure 4D). Indeed, *neg_1* is lower for the high impulsivity group, suggesting that a negative outcome at *t*−1 has greater influence in more impulsive subjects' switching behavior.

To investigate whether the switching measures above are related to specific dimensions of impulsivity, we performed the correlation analyses described above with each subscale score. Significant correlation with *p*(*a|ab*) was strong in Sensation Seeking (Pearson *r* = −0.29, *p* = 0.038 uncorrected); there was no correlation with the other subscales. There was a significant but weak correlation of *p*(*stay*|- - -) with Positive Urgency (Spearman ρ = −0.24, *p* = 0.045) and Sensation Seeking (Pearson *r* = −0.24, *p* = 0.049) (see Supplementary Table 1 for all correlations between subdimensions and key behavioral measures).

We also conducted five additional logistic mixed effects models with outcome interactions, each using an S-UPPS-P subscale score in place of total impulsivity score (see Supplementary Table 2 for interactions between each subscale and *neg_1*). The impulsivity subscale interaction with *neg_1* was strongest for Positive Urgency (B = −0.12, SE = 0.025, and *p* < 0.05 uncorrected) and Sensation Seeking (B = −0.10, SE = 0.024, and *p* < 0.05), weaker in Negative Urgency (B = −0.071, SE = 0.025, and *p* < 0.05) and Lack of Perseverance (B = 0.048, SE = 0.024, and *p* < 0.05), and not significant for Lack of Premeditation (B = 0.0019, SE = 0.025, and *p* = 0.94).

While *ad hoc* and exploratory, these more fine-grained, trial history-dependent analyses reveal effects of impulsivity on switching behavior under more specific sequences of trial history and survive multiple comparison correction. They indicate that the effect of impulsivity in probabilistic switching may be subtle, and related to multi-trial decision-making sequences, rather than to simple reactions to a single choice and outcome.

### Computational Modeling Captures Group-Level Behavior, but Not Impulsivity Effects

**Figure 5:**
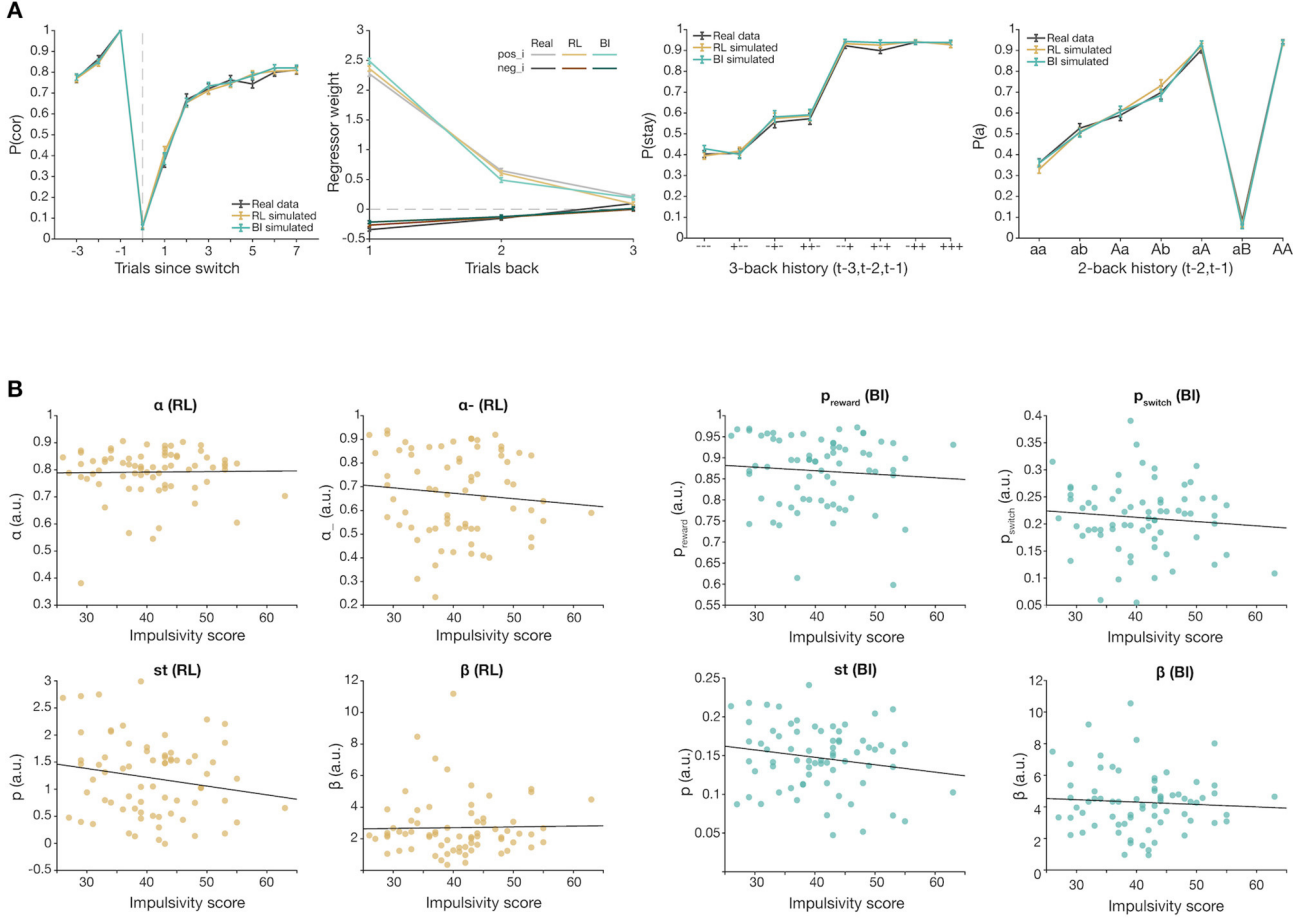
Computational modeling results of best-fitting models, RL (brown) and BI (teal). **(A)**. Comparison of behavior simulated from best fitting models RL and BI, and real participant behavior (black). Both models capture group-level switching behavior. **(B)** Total impulsivity score vs. fit parameter values of RL (left) and BI (right) (all *p*s > 0.05).

We used computational modeling to capture the underlying cognitive processes driving task behavior. We fit a reinforcement learning model (RL) with four parameters (learning rates α and α_−_, sticky parameter *st*, and inverse temperature β), and a Bayesian inference model (BI) with four parameters (*p*_*reward*_, *p*_*switch*_, sticky parameter *st*, and inverse temperature β). WAIC score comparison showed that the chosen RL model was the best performing model within the RL model family. Both models successfully captured group-level switching behavior (Figure 5A), as well as other patterns of behaviors. Parameter recovery was successful for each model. However, there was no significant correlation between overall impulsivity score and fit parameter values in either model (Figure 5B), and validation simulations were not able to replicate observed group differences, highlighting a failure in capturing individual differences in the model, despite satisfactory group-level modeling.

## Discussion

The purpose of our study was to elucidate the relationship of trait-like impulsivity with decision-making in an uncertain, volatile environment, and to consider whether these effects varied for specific dimensions of impulsivity. Based on previous studies, we had predicted that more impulsive participants would show poorer performance, and in particular a higher tendency to switch after a negative outcome (48). While our results did not support our a *priori* predictions, we did observe an effect of impulsivity on switching behavior in the context of multiple past negative outcomes. That is, more impulsive participants were more likely to switch their past trial's choice after three consecutive unrewarded outcomes. This suggests that impulsivity may play a role in how negative outcomes, and in particular the compounded effects of multiple negative outcomes in sequence, drive decision-making. A simple interpretation of this result is that impulsivity reduces the amount of evidence needed to switch after negative outcomes. Obtaining three negative outcomes in a row, however, could be the result of different choice strategies (such as sticking with the previously correct choice long after a reversal, or switching away from the correct choice after an unrewarded correct choice), which should be interpreted differently. Another interpretation could be that participants with different levels of impulsivity may have understood the task differently; however, we think this interpretation is unlikely, given that the behavior of participants who were more impulsive was comparable in most measures to that of those less impulsive (Figure 3).

The results of the aB analysis suggest a slightly different interpretation. We found that more impulsive participants were more likely to return to an unrewarded action if their switch was also unrewarded, showing less “commitment” to testing out the other option after a switch. While both actions could be unrewarded back-to-back, one action must be correct; because correct reversals are always rewarded, an unrewarded switch indicates that action was correctly answered (but simply unrewarded due to stochasticity). Thus, impulsivity may contribute to misreading noise as a signal for reversal, leading more impulsive individuals to produce multiple switches, and to less extended periods of “hypothesis testing” after a switch. Although impulsivity did not relate to overall performance or simple win-stay lose-switch behavior, our results did reveal an increased sensitivity to negative outcome in the context of multi-trial sequences of choices and outcomes, making it likely that this relates to participants' strategies in the task. This is consistent with previous findings of higher switching behavior following unrewarded stimuli in a task involving learning to detect hidden probabilistic rewards (48).

We note that it is not necessarily surprising to observe a lack of significant correlation in overall task performance with impulsivity, despite more subtle differences in strategy brought to light with more advanced analyses. In the “ab” context, high impulsivity participants are more likely to switch back and forth between actions, but this switching is not necessarily detrimental: in the case where action *a* was correct but unrewarded, returning to *a*, instead of staying with the incorrect *b*, would increase the chances of obtaining a reward. Thus, if interactions between impulsivity and negative outcomes at *t–*1 are driven by a double switch in “ab” that does not always delay point accrual, more impulsive participants would still be able to progress through the task at a pace comparable to their less impulsive counterparts, whose strategy may be to stay with an unrewarded choice to confirm a true reversal has occurred, then switch and fully commit to the other action. Staying with the unrewarded choice prevents immediate reward but ensures later reward in the long run, in line with the general idea that impulsivity is associated with immediate reward-seeking, even at the risk of incurring long-term negative consequences. A Spearman correlation of impulsivity score and the average number of reversals per block was not significant (ρ = 0.079, *p* = 0.52), indicating that both strategies are carried out by comparable numbers of trials, which also serve as a proxy for performance (task progression is determined by the participant).

The absence of a main effect of impulsivity on our a priori measures of overall task performance was unexpected, given prior findings. For example, contrary to Sali et al. (48), we found that more impulsive participants were equally likely as less impulsive participants to stay when the previous trial was rewarded and equally likely to switch when the previous trial was unrewarded. Indeed, past research shows mixed results, with some reward-based learning studies revealing association of self-reported impulsivity with reward-based task behavior (45), whereas others found no such association (70). One main limitation of our study is our reliance on undergraduate students, who showed relatively low S-UPPS-P scores [see Supplementary Figure S2 for score distributions of overall impulsivity and subscales; cf, UPPS-P mean scores from (33)]. The score distributions of both impulsivity groups in our study (low: *M* = 34.4, SD = 4.22; high: *M* = 47.0, SD = 5.08) are consistently lower than those of other studies, such as Fernández-Serrano et al. (46), who used a 59-item UPPS-P scale (low: *M* = 111.7, SD = 13.3; high: *M* = 141.7, SD = 16.4), and Xiao et al. (42), who used a 45-item UPPS scale (low: *M* = 100.3, 11.7; *M* = 110.7, SD = 11.7). Our range of scores was similar to Nebe et al. (70) who used the BIS-11 scale, but also found no significant effect of impulsivity; studies that have found associations with impulsivity have distributions spanning a higher range for more impulsive scores (37, 45, 48). Previous studies that have reported effects of impulsivity on reward-learning tasks included high-risk groups, such as those with substance use or gambling concerns. In the broader literature, correlations of impulsivity with other laboratory tasks, such as response inhibition, are systematically higher in clinical studies, which tend to have more severe impulsivity concerns, as compared to community or student samples (7). Beyond the sample, cross-study discrepancies could relate to the difference in experimental protocol. Sali et al. (48) used a probabilistic task with binary outcomes, as we did, but the link between impulsivity and “behavioral stability” (win-stay/lose-switch behavior) was observed in the stable portion of the task with sparse reward outcomes, which may, again, have elicited very different strategies. Many earlier studies [e.g., (37)] focused on the role of impulsivity in addiction, and for that reason, used gambling-like protocols, including variants of the Iowa gambling task or other gambling-like reversal tasks. These protocols require participants to integrate uncertain outcomes across multiple possible values, which may elicit different processes than in our task. Although our null effects conflict with some previous work, it is important to note that others have found that the link between impulsivity and probabilistic reversal learning is contingent on other variables, such as a dopaminergic drug manipulation (71).

Overall, subtly different reward learning tasks may elicit vastly different cognitive strategies. In large-scale work, individual differences in the Reinforcement Learning Working Memory task (RLWM) do not mirror those in the probabilistic switching task (72). Indeed, we chose these two tasks to index fairly separate and complementary influences of learning and executive function in adaptive behavior. Findings here highlight the import of considering this in relation to impulsivity. More specifically, while we found that impulsivity affected participants' strategy in the probabilistic reversal learning task, we found no effect of impulsivity in RLWM (see Supplementary Results, Supplementary Figure S5). In RLWM, participants learn stimulus-action associations from reward outcomes across blocks varying in set-size; learning varies as a function of set-size due to differences in use of reinforcement learning, reliance on working memory, and interactions between the two cognitive systems (49).

We had hypothesized that higher impulsivity would predict lower overall task performance, and drawing on theory about the import of executive function in interaction with reward-seeking, we predicted impulsivity effects would be specific to high set-size blocks with multiple stimuli to learn. However, our results showed no effects of impulsivity on performance in any set size, during either learning or retention. Despite the numerically better asymptotic training performance on ns = 6 blocks of the high impulsivity group (*M* = 0.867, SE = 0.022) compared to that of the low impulsivity group (*M* = 0.819, SE = 0.022), there was no significant effect of impulsivity [Welch's *t*-test, *t*_(77)_ = −1.53, *p* = 0.129; Hedges' *g* = −0.343]. Further tests on smaller set sizes and other behavioral measures consistently indicated no effect of impulsivity. Computational modeling also revealed no differences in the two processes supporting learning. In particular, there was no significant correlation of the parameter WM capacity K, our measure of working memory, and impulsivity. Our findings fit with a growing body of work that WM is not consistently tied to impulsivity [cf. (39)]. Future work would do well to consider different forms of executive function in interaction with reward-seeking, such as response inhibition. WM influences are also likely to vary substantively across different tasks, thus interacting with impulsivity differently. Stated differently, impulsivity effects might emerge in conditions with high working memory demand that differ from the ones we tested here (i.e., uncertainty in the form of probabilistic feedback, or when reversals occur).

Our computational modeling results were also mixed. On the positive side, both models could adequately capture group-level behavior in the probabilistic reversal side. However, both models failed to capture individual differences as indexed by impulsivity: simulated model behavior with fit parameters did not reproduce impulsivity effects, and individual fit parameters did not correlate with impulsivity. Our RL model parameterized feedback-dependent learning (α, α_−_), counterfactual updating of unchosen actions, value sensitivity (β), and sticky behavior (st). Even though Q-learning should slowly update expected values of actions, the additional parameters enabled cognitive flexibility and learning even from stochastic feedback. Our BI model differed from RL in the use of p_reward_ and p_switch_ for Bayesian inference, but also incorporated the same β and st parameters. Similarly, it was also successful in capturing real switching behavior and asymptotic performance (Figure 5A; Supplementary Figure S3). Indeed, previous work using computational models to describe behavior in a binary-choice RL task found associations between high impulsivity, as measured by BIS-11, and two parameters encoding uncertainty-dependent mechanisms of belief updates (73). In contrast, we did not find significant correlations of our fitted model parameters with impulsivity; that neither model could capture the specific effect of impulsivity suggests that both may lack some parameter specifically accounting for a noise vs. reversal signal judgment. One possibility is that impulsivity reflects multiple cognitive factors, which may differ across individuals. This modeling limitation warrants future investigation.

One of our goals was to examine specific dimensions of impulsivity, particularly given previous work that suggested that Negative Urgency was more powerfully tied to perseverative errors on response reversal task (46), and that Negative Urgency and (lack of) Perseverance were tied to diminished reward learning on the IGT (42), and that (lack of) Premeditation was correlated with outcomes in a different gambling task (41). Our findings did not mirror these effects, in that we found significant effects of Positive Urgency and Sensation Seeking on p(*stay*|- - -) and Sensation Seeking on *p*(*a|ab*), but not Negative Urgency, Perseverance, or Premeditation. In one meta-analysis of delay of gratification, researchers observed the Urgency scale was only correlated with delay of gratification in those studies using contingent delay of gratification (i.e., where actual money was involved), and not in those using hypothetical rewards (6).

## Conclusion

Our study aimed to understand the role of impulsivity, quantified using the S-UPPS-P scale, in learning and decision-making in a probabilistic switching task. We tested effects using a task that allows us to measure and think about response inhibition, and our computational models successfully captured group-level behavior. Nonetheless, we observed null effects of impulsivity in our a *priori* analyses of general behaviors such as task performance and switching behavior. In exploratory analyses, we also could not identify effects of impulsivity in computational models. Rather, exploratory analyses indicated that impulsivity plays a nuanced role in the judgment of negative outcomes resulting from stochastic noise vs. reversal signal that motivate switching behavior. Given the import of impulsivity for many psychopathology outcomes, expanding approaches to understanding behavioral and computational aspects of impulsivity is an important goal.

## Data Availability Statement

The raw data supporting the conclusions of this article will be made available by the authors, without undue reservation.

## Ethics Statement

The studies involving human participants were reviewed and approved by UC Berkeley Committee for Protection of Human Subjects. The patients/participants provided their written informed consent to participate in this study.

## Author Contributions

AZ and DML collected the data. AZ, DML, and AC analyzed the data. AZ and AC performed the computational modeling. AZ wrote the first draft of the manuscript. All authors wrote sections of the manuscript, contributed to conception and design of the study, manuscript revision, read, and approved the submitted version.

## Funding

AC was supported by NIH R01MH119383 and NSF2020844. SJ was supported by NIH R01MH110477.

## Conflict of Interest

The authors declare that the research was conducted in the absence of any commercial or financial relationships that could be construed as a potential conflict of interest.

## Publisher's Note

All claims expressed in this article are solely those of the authors and do not necessarily represent those of their affiliated organizations, or those of the publisher, the editors and the reviewers. Any product that may be evaluated in this article, or claim that may be made by its manufacturer, is not guaranteed or endorsed by the publisher.

## References

[1] Raio CM Konova AB Otto AR. Trait impulsivity and acute stress interact to influence choice and decision speed during multi-stage decision-making. Sci Rep. (2020) 10:7754. 10.1038/s41598-020-64540-0 32385327 PMC7210896

[2] Berg JM Latzman RD Bliwise NG Lilienfeld SO. Parsing the heterogeneity of impulsivity: a meta-analytic review of the behavioral implications of the UPPS for psychopathology. Psychol Assess. (2015) 27:1129–46. 10.1037/pas0000111 25822833

[3] Bechara A. Decision making, impulse control and loss of willpower to resist drugs: a neurocognitive perspective. Nat Neurosci. (2005) 8:1458–63. 10.1038/nn1584 16251988

[4] Carver CS Johnson SL Joormann J. Two-mode models of self-regulation as a tool for conceptualizing effects of the serotonin system in normal behavior and diverse disorders. Curr Dir Psychol Sci. (2009) 18:195–9. 10.1111/j.1467-8721.2009.01635.x 20161026 PMC2749682

[5] Nigg JT. Annual Research Review: on the relations among self-regulation, self-control, executive functioning, effortful control, cognitive control, impulsivity, risk-taking, and inhibition for developmental psychopathology. J Child Psychol Psychiatry. (2017) 58:361–83. 10.1111/jcpp.12675 28035675 PMC5367959

[6] Sharma L Markon KE Clark LA. Toward a theory of distinct types of “impulsive” behaviors: a meta-analysis of self-report and behavioral measures. Psychol Bull. (2014) 140:374–408. 10.1037/a0034418 24099400

[7] Johnson SL Tharp JA Peckham AD Sanchez AH Carver CS. Positive urgency is related to difficulty inhibiting prepotent responses. Emotion. (2016) 16:750–9. 10.1037/emo0000182 27064288 PMC4980191

[8] Gunn RL Finn PR. Impulsivity partially mediates the association between reduced working memory capacity and alcohol problems. Alcohol. (2013) 47:3–8. 10.1016/j.alcohol.2012.10.003 23200800 PMC3545083

[9] Hinson JM Jameson TL Whitney P. Impulsive decision making and working memory. J Exp Psychol Learn Mem Cogn. (2003) 29:298–306. 10.1037/0278-7393.29.2.298 12696817

[10] Patton JH Stanford MS Barratt ES. Factor structure of the Barratt impulsiveness scale. J Clin Psychol. (1995) 51:768–74. 10.1002/1097-4679(199511)51:6<768::AID-JCLP2270510607>3.0.CO 8778124

[11] Berlin HA Rolls ET Iversen SD. Borderline personality disorder, impulsivity, and the orbitofrontal cortex. Am J Psychiatry. (2005) 162:2360–73. 10.1176/appi.ajp.162.12.2360 16330602

[12] Day AM Metrik J Spillane NS Kahler CW. Working memory and impulsivity predict marijuana-related problems among frequent users. Drug Alcohol Depend. (2013) 131:171–4. 10.1016/j.drugalcdep.2012.12.016 23312340 PMC3626751

[13] Lopez-Vergara HI Jackson KM Meshesha LZ Metrik J. Dysregulation as a correlate of cannabis use and problem use. Addict Behav. (2019) 95:138–44. 10.1016/j.addbeh.2019.03.010 30913511 PMC8453490

[14] Mueller SM Schiebener J Stöckigt G Brand M. Short- and long-term consequences in decision-making under risk: immediate feedback about long-term prospects benefits people tending to impulsive processing. J Cogn Psychol. (2017) 29:217–39. 10.1080/20445911.2016.1245660

[15] Whitney P Jameson T Hinson JM. Impulsiveness and executive control of working memory. Pers Individ Differ. (2004) 37:417–28. 10.1016/j.paid.2003.09.013

[16] Zeng H Lee TMC Waters JH So K-F Sham PC Schottenfeld RS. Impulsivity, cognitive function, and their relationship in heroin-dependent individuals. J Clin Exp Neuropsychol. (2013) 35:897–905. 10.1080/13803395.2013.828022 24040894

[17] Manning V Teoh HC Guo S Wong KE Li T-K. Executive functioning in Asian pathological gamblers. Int Gambl Stud. (2013) 13:403–16. 10.1080/14459795.2013.829516

[18] Lempert KM Pizzagalli DA. Delay discounting and future-directed thinking in anhedonic individuals. J Behav Ther Exp Psychiatry. (2010) 41:258–64. 10.1016/j.jbtep.2010.02.003 20219184 PMC2862767

[19] Visser TAW Bender AD Bowden VK Black SC Greenwell-Barnden J Loft S. Individual differences in higher-level cognitive abilities do not predict overconfidence in complex task performance. Conscious Cogn. (2019) 74:102777. 10.1016/j.concog.2019.102777 31271910

[20] Brooks SJ Wiemerslage L Burch K Maiorana S Cocolas E Schiöth H. The impact of cognitive training in substance use disorder: the effect of working memory training on impulse control in methamphetamine users. Psychopharmacology. (2017) 234:1911–21. 10.1007/s00213-017-4597-6 28324119 PMC5486910

[21] Wanmaker S Leijdesdorff SMJ Geraerts E van de Wetering BJM Renkema PJ Franken IHA. The efficacy of a working memory training in substance use patients: a randomized double-blind placebo-controlled clinical trial. J Clin Exp Neuropsychol. (2017) 40:473–86. 10.1080/13803395.2017.1372367 28933254

[22] Carver CS Johnson SL Joormann J Kim Y Nam JY. Serotonin transporter polymorphism interacts with childhood adversity to predict aspects of impulsivity. Psychol Sci. (2011) 22:589–95. 10.1177/0956797611404085 21460340

[23] Cyders MA Smith GT. Mood-based rash action and its components: positive and negative urgency. Pers Individ Differ. (2007) 43:839–50. 10.1016/j.paid.2007.02.008

[24] Whiteside SP Lynam DR Miller JD Reynolds SK. Validation of the UPPS impulsive behaviour scale: a four-factor model of impulsivity. Eur J Pers. (2005) 19:559–74. 10.1002/per.55628118729

[25] Whiteside SP Lynam DR. The five factor model and impulsivity: using a structural model of personality to understand impulsivity. Pers Individ Differ. (2001) 30:669–89. 10.1016/S0191-8869(00)00064-7

[26] Carver CS Johnson SL Timpano KR. Toward a functional view of the P factor in psychopathology. Clin Psychol Sci. (2017) 5:880–9. 10.1177/2167702617710037 29057170 PMC5646702

[27] Cyders MA Smith GT. Emotion-based dispositions to rash action: positive and negative urgency. Psychol Bull. (2008) 134:807–28. 10.1037/a0013341 18954158 PMC2705930

[28] Billieux J Heeren A Rochat L Maurage P Bayard S Bet R. Positive and negative urgency as a single coherent construct: Evidence from a large-scale network analysis in clinical and non-clinical samples. J Pers. (2021) 89:1252–62. 10.1111/jopy.12655 34114654 PMC9292904

[29] Noël X Saeremans M Kornreich C Bechara A Jaafari N Fantini-Hauwel C. On the processes underlying the relationship between alexithymia and gambling severity. J Gambl Stud. (2017) 34:1049–66. 10.1007/s10899-017-9715-1 28866795

[30] Mallorquí-Bagué N Tolosa-Sola I Fernández-Aranda F Granero R Fagundo AB Lozano-Madrid M. Cognitive deficits in executive functions and decision-making impairments cluster gambling disorder sub-types. J Gambl Stud. (2018) 34:209–23. 10.1007/s10899-017-9724-0 29058168

[31] Gunn RL Finn PR. Applying a dual process model of self-regulation: the association between executive working memory capacity, negative urgency, and negative mood induction on pre-potent response inhibition. Pers Individ Differ. (2015) 75:210–5. 10.1016/j.paid.2014.11.033 25530648 PMC4269241

[32] Rochat L Beni C Annoni J-M Vuadens P Van der Linden M. How inhibition relates to impulsivity after moderate to severe traumatic brain injury. J Int Neuropsychol Soc. (2013) 19:890–8. 10.1017/S1355617713000672 23816263

[33] Wardell JD Quilty LC Hendershot CS. Impulsivity, working memory, and impaired control over alcohol: a latent variable analysis. Psychol Addict Behav. (2016) 30:544–54. 10.1037/adb0000186 27269291 PMC5266597

[34] Li J Weidacker K Mandali A Zhang Y Whiteford S Ren Q. Impulsivity and craving in subjects with opioid use disorder on methadone maintenance treatment. Drug Alcohol Depend. (2021) 219:108483. 10.1016/j.drugalcdep.2020.108483 33385690

[35] Burkard C Rochat L Emmenegger J Van der Linden A-CJ Gold G Van der Linden M. Implementation intentions improve prospective memory and inhibition performances in older adults: the role of visualization. Appl Cogn Psychol. (2014) 28:640–52. 10.1002/acp.3046

[36] Keye D Wilhelm O Oberauer K. Structure and correlates of the german version of the brief UPPS impulsive behavior scales. Eur J Psychol Assess. (2009) 25:175–85. 10.1027/1015-5759.25.3.175

[37] Franken IHA van Strien JW Nijs I Muris P. Impulsivity is associated with behavioral decision-making deficits. Psychiatry Res. (2008) 158:155–63. 10.1016/j.psychres.2007.06.002 18215765

[38] Penolazzi B Leone L Russo PM. Individual differences and decision making: when the lure effect of gain is a matter of size. PLoS One. (2013) 8:e58946. 10.1371/journal.pone.0058946 23484058 PMC3590131

[39] Bjork JM Hommer DW Grant SJ Danube C. Impulsivity in abstinent alcohol-dependent patients: relation to control subjects and type 1-/type 2-like traits. Alcohol. (2004) 34:133–150. 10.1016/j.alcohol.2004.06.012 15902907

[40] Niv Y. Reinforcement learning in the brain. Journal of Mathematical Psychology. (2009) 53:139–54. 10.1016/j.jmp.2008.12.005

[41] Zermatten A Van Der Linden M D'Acremont M Jermann F Bechara A. Impulsivity and decision making. J Nerv Ment Dis. (2005) 193:647–50. 10.1097/01.nmd.0000180777.41295.65 16208159

[42] Xiao L Bechara A Grenard LJ Stacy WA Palmer P Wei Y. Affective decision-making predictive of Chinese adolescent drinking behaviors. J Int Neuropsychol Soc. (2009) 15:547–57. 10.1017/S1355617709090808 19573273 PMC3626262

[43] Berlin HA Rolls ET Kischka U. Impulsivity, time perception, emotion and reinforcement sensitivity in patients with orbitofrontal cortex lesions. Brain. (2004) 127:1108–26. 10.1093/brain/awh135 14985269

[44] Clatworthy PL Lewis SJG Brichard L Hong YT Izquierdo D Clark L. Dopamine release in dissociable striatal subregions predicts the different effects of oral methylphenidate on reversal learning and spatial working memory. J Neurosci. (2009) 29:4690–6. 10.1523/JNEUROSCI.3266-08.2009 19369539 PMC6665353

[45] Vanes LD van Holst RJ Jansen JM van den Brink W Oosterlaan J Goudriaan AE. Contingency learning in alcohol dependence and pathological gambling: Learning and unlearning reward contingencies. Alcoholism Clin Exp Res. (2014) 38:1602–10. 10.1111/acer.12393 24821534 PMC4171748

[46] Fernández-Serrano MJ Perales JC Moreno-López L Pérez-García M Verdejo-García A. Neuropsychological profiling of impulsivity and compulsivity in cocaine dependent individuals. Psychopharmacology. (2012) 219:673–83. 10.1007/s00213-011-2485-z 21922168

[47] Paliwal S Petzschner FH Schmitz AK Tittgemeyer M Stephan KE. A model-based analysis of impulsivity using a slot-machine gambling paradigm. Front Hum Neurosci. (2014) 8:428. 10.3389/fnhum.2014.00428 25071497 PMC4080386

[48] Sali AW Anderson BA Yantis S. Reinforcement learning modulates the stability of cognitive control settings for object selection. Front Integr Neurosci. (2013) 7:95. 10.3389/fnint.2013.00095 24391557 PMC3866588

[49] Collins AGE. The tortoise and the hare: interactions between reinforcement learning and working memory. J Cogn Neurosci. (2018) 30:1422–32. 10.1162/jocn_a_01238 29346018

[50] Collins AGE Frank MJ. How much of reinforcement learning is working memory, not reinforcement learning? A behavioral, computational, and neurogenetic analysis: working memory in reinforcement learning. Eur J Neurosci. (2012) 35:1024–35. 10.1111/j.1460-9568.2011.07980.x 22487033 PMC3390186

[51] Tai L-H Lee AM Benavidez N Bonci A Wilbrecht L. Transient stimulation of distinct subpopulations of striatal neurons mimics changes in action value. Nat Neurosci. (2012) 15:1281–9. 10.1038/nn.3188 22902719 PMC3951287

[52] de Leeuw JR. jsPsych: a JavaScript library for creating behavioral experiments in a Web browser. Behav Res Methods. (2015) 47:1–12. 10.3758/s13428-014-0458-y 24683129

[53] Stewart CA Turner G Vaughn M Gaffney NI Cockerill TM Foster I. Jetstream: a self-provisioned, scalable science and engineering cloud environment. In: Proceedings of the 2015 XSEDE Conference on Scientific Advancements Enabled by Enhanced Cyberinfrastructure - XSEDE '15 (New York, NY). (2015). p. 1–8.

[54] Towns J Cockerill T Dahan M Foster I Gaither K Grimshaw A. XSEDE: accelerating scientific discovery. Comput Sci Eng. (2014) 16:62–74. 10.1109/MCSE.2014.80

[55] Cyders MA Littlefield AK Coffey S Karyadi KA. Examination of a short English version of the UPPS-P Impulsive Behavior Scale. Addict Behav. (2014) 39:1372–6. 10.1016/j.addbeh.2014.02.013 24636739 PMC4055534

[56] The The MathWorks, Inc. MATLAB and Statistics Toolbox (Release 2020a) [Computer software] (2020).

[57] R Core Team. R: A Language and Environment for Statistical Computing. Vienna: R Foundation for Statistical Computing (2021).

[58] Bates D Mächler M Bolker B Walker S. Fitting linear mixed-effects models using lme4. J Stat Softw. (2015) 67:1–48. 10.18637/jss.v067.i01

[59] Eckstein MK Master SL Dahl RE Wilbrecht L Collins AGE. Reinforcement learning and Bayesian inference provide complementary models for the unique advantage of adolescents in stochastic reversal [Preprint]. Neuroscience. (2020). 10.1101/2020.07.04.187971 PMC9108470 35537273

[60] Gershman SJ Uchida N. Believing in dopamine. Nat Rev Neurosci. (2019) 20:703–14. 10.1038/s41583-019-0220-7 31570826 PMC7472313

[61] Katahira K. How hierarchical models improve point estimates of model parameters at the individual level. J Math Psychol. (2016) 73:37–58. 10.1016/j.jmp.2016.03.007

[62] Stan Development Team. Stan Modeling Language Users Guide Reference Manual (2.26.1.) [Computer software]. (2021). Available online at: https://mc-stan.org (accessed June 01, 2021).

[63] Baribault B Collins A. Troubleshooting Bayesian Cognitive Models: A Tutorial With Matstanlib (2021). 10.31234/osf.io/rtgew PMC10522800 36972080

[64] Lau B,. (2017) *MatlabStan: The MATLAB interface to Stan (2.15.1.0) [Computer software]*. Available online at: https://mc-stan.org/users/interfaces/matlab-stan (accessed June 01, 2021).

[65] Vehtari A Gelman A Gabry J. Practical Bayesian model evaluation using leave-one-out cross-validation and WAIC. Stat Comput. (2017) 27:1413–32. 10.1007/s11222-016-9696-4

[66] Wilson RC Collins AG. Ten simple rules for the computational modeling of behavioral data. ELife. (2019) 8:e49547. 10.7554/eLife.49547 31769410 PMC6879303

[67] Watanabe S. A widely applicable Bayesian information criterion. J Mach Learn Res. (2013) 14:867–97.

[68] Gelman A Hwang J Vehtari A. Understanding predictive information criteria for Bayesian models. Stat Comput. (2014) 24:997–1016. 10.1007/s11222-013-9416-2

[69] Beron C Neufeld S Linderman S Sabatini B. Efficient and stochastic mouse action switching during probabilistic decision making [Preprint]. Neuroscience. (2021). 10.1101/2021.05.13.444094 PMC9169659 35385355

[70] Nebe S Kroemer NB Schad DJ Bernhardt N Sebold M Müller DK. No association of goal-directed and habitual control with alcohol consumption in young adults: alcohol use and learning. Addict Biol. (2018) 23:379–93. 10.1111/adb.12490 28111829

[71] Cools R Sheridan M Jacobs E D'Esposito M. Impulsive personality predicts dopamine-dependent changes in frontostriatal activity during component processes of working memory. J Neurosci. (2007) 27:5506–14. 10.1523/JNEUROSCI.0601-07.2007 17507572 PMC6672352

[72] Eckstein MK Master SL Xia L Dahl RE Wilbrecht L Collins AGE. Learning rates are not all the same: the interpretation of computational model parameters depends on the context [Preprint]. Neuroscience. (2021). 10.1101/2021.05.28.446162 PMC9635876 36331872

[73] Lim MSM Jocham G Hunt LT Behrens TEJ Rogers RD. Impulsivity and predictive control are associated with suboptimal action-selection and action-value learning in regular gamblers. Int Gambl Stud. (2015) 15:489–505. 10.1080/14459795.2015.1078835 27274706 PMC4890653

