# Supplementary material for: Impulsivity Relates to Multi-Trial Choice Strategy in Probabilistic Reversal Learning
Source: Front Psychiatry. 2022 Mar 14;13:800290. doi: 10.3389/fpsyt.2022.800290 (PMC8964258; doi:10.3389/fpsyt.2022.800290)
Supplement: Supplementary file 1 [file Data_Sheet_1.docx]

**Supplementary Information**

**SUPPS-P Questionnaire**

Participants were asked to complete the Short UPPS-P questionnaire, a 20-item version of the original 59-item UPPS-P (Cyders et al., 2014). The items were measured on a scale of 1 (agree strongly) to 4 (disagree strongly). Twelve of the 20-items were reversed scored, such that a response of 1 (agree strongly) was actually a 4 (disagree strongly), and vice versa. The questionnaire consists of five subscales which measure subcomponents of impulsivity: negative urgency, lack of premeditation, lack of perseverance, sensation seeking, and positive urgency. Participants were asked to indicate how much they agreed or disagreed with each statement presented.

**Probabilistic Switching Task instructions**

The following instructions were provided one screen at a time, interleaved with practice of each specific instruction (Eckstein et al., submitted).

1. This experiment requires your full attention. Before we begin, please maximize your browser window and turn off all notifications (Facebook, texts, etc.) on both your phone and computer. When you're ready, you can press any key to start.
2. Try to collect all the coins.
3. The coins are hidden in magical boxes, like this one.
4. Can you open the box to see if it has a coin? Press the J key to open the box!
5. In the game, there will be two boxes, but only one is magical. Try finding the magical box. The J key opens the left box and the K key opens the right box.
6. Sometimes, the magical box switches sides. Try finding the magical box again!
7. Sometimes, the magical box may not have a coin. This makes it harder to find. Can you still find the magical box?
8. Now the real game will begin. Try your best to find as many coins as you can!

**Supplementary methods: RLWM task**

*Task design:*

As with the probabilistic switching task, participants completed the reinforcement learning and working memory (RLWM) task remotely from their own computer; the task was written using the Javascript library jsPsych, and the website was hosted by Jetstream. The RWLM task consisted of a learning phase and a testing phase (Collins, 2018).

In the learning phase, participants saw images presented individually on the screen, and were instructed to learn the correct key for the image. They were told that only one key out of three (“J”, ”K”, and ”L”) was correct for each stimulus, and that they would receive a point for each correct key press. Contrary to the probabilistic reversal task, outcomes were deterministic, and correct choice for a given stimulus did not change during a block. Participants completed practice trials as part of their instructions, and were allowed to repeat the instructions if needed.

Participants completed 12 independent learning blocks with new images each coming from a different semantic category (e.g. colors, animals, vegetables). Blocks varied in set size, or the number of categorically related images to learn; there were 2 blocks of set size 2, 3 of set size 3, 2 blocks of set size 4, 3 blocks of set size 5, and 2 blocks of set size 6. At the start of the block, participants were given 10 seconds to identify all the images to learn in the block. In a given trial, the stimulus image was shown on the center of the screen for 1.5 seconds, during which participants were expected to press a key. Deterministic feedback was presented for 0.5 s: a “+1” in green for a correct action, or “+0” in red for an incorrect action; if no response was made, “You took too long to respond!” was shown on the screen. Finally, a fixation cross was shown for 0.5 s to signal the start of the next trial. Each stimulus in a set size was presented 13 times, and the block trial sequence was pre-determined to counterbalance delays between presentations within each stimulus. At the end of the block, participants were shown their final point tally, and given a 30 second break before the start of the next trial.

Following a block of the probabilistic switching task, participants returned to RLWM to complete the testing phase, which evaluated retention of the learned stimulus-action associations during the learning task. During the testing phase, participants saw all 48 independent stimuli again, each shown three times, and were asked to press the correct key based on what they learned in the learning phase. No feedback was presented following action selection, though feedback was still given for missed responses. The testing phase contained 144 trials, and the stimulus presentation order was shuffled within 48 trials.

*Behavioral and statistic analysis:*

Behavioral and statistical analysis of the RLWM task was conducted in MATLAB. For the learning phase, we used mnrfit to fit a multinomial logistic regression on each participant’s data to predict accuracy by set size, delay between correctly answered stimuli, and stimulus reward history (Master et al., 2020). For the testing phase, we fit a multinomial logistic regression to predict accuracy by set size, asymptotic learning phase performance for a given stimulus, and block (Collins, 2018).

*Computational modeling:*

To uncover underlying cognitive processes driving the RLWM task behavior, we fit a series of learning models to the learning and testing phase (Collins, 2018; Collins & Frank, 2012) to the data. Models including a mixture of two processes (RL and WM) fit better than models with a single process. The winning RLWM model mixed features of Master et al 2019 and Collins 2018, and contained 7 parameters: learning rate (α), choice noise (ε), negative learning rate bias (bias), working memory capacity (K), weight (ρ), and decay (ϕ), and test phase β.

This RLWM model assumes that choice is a mixture of RL and WM policies:

$$P\left( a | s \right)=w*p_{WM}\left( a | s \right)+\left( 1-w \right)*p_{RL}(a|s)$$

Where w reflects the WM contributions: $w= \rho*min(1,\frac{K}{setsize})$, and ρ is the WM confidence prior.

The RL policy is an epsilon-softmax transform of RL Q-values:

$$p_{RL}\left( a | s \right)=\frac{\varepsilon}{3}+\left( 1-\varepsilon\right)*softmax(\beta*Q(s,a))$$

The WM policy is an epsilon-softmax transform of WM weights:

$$p_{WM}\left( a | s \right)=\frac{\varepsilon}{3}+\left( 1-\varepsilon\right)*softmax(\beta*W(s,a))$$

$\beta$ is fixed to 50, to represent perfect WM retention one-trial back, and because previous research has shown that this parameter is poorly recoverable when free.

Q-values were initialized to neutral value $Q_{0}=\frac{1}{3}$, and updated according to the standard delta rule:

$$Q_{t+1}\left( s,a \right)=Q_{t}\left( s,a \right)+\alpha*(r_{t}-E_{t}\left( s,a \right))$$

For negative outcomes, learning rate was down-weighted by the bias parameter.

To capture interference of WM in RL learning, following Collins 2018, the expected outcome $E_{t}\left( s,a \right)$ was not $Q_{t}\left( s,a \right)$ as usual, but the mixture of $Q$ and $W$:

$$E_{t}\left( s,a \right)=w*W\left( s,a \right)+\left( 1-w \right)*Q(s,a)$$

WM weights were initialized to neutral value $W_{0}=\frac{1}{3}$, and updated in two ways.

For WM encoding, positive outcome updated weights with learning rate with learning rate 1, such that $W_{t+1}\left( s,a \right) =1$; negative outcomes updated weights with learning rate 1*bias such that $W_{t+1}\left( s,a \right) =W_{t}\left( s,a \right)+bias*\left( 0-W_{t}\left( s,a \right) \right)$.

To capture WM forgetting, all other stimuli weights were decayed at each trials according to $W_{t+1}=W+\phi*(W_{0}-W_{t})$.

The model fitting process used maximum likelihood estimation, following Wilson & Collins 2019. The approach was validated, and the model captured behavior appropriately.

**Supplementary Results: RLWM**

We analyzed data from 79 participants (following exclusions for poor performance). Consistent with our results for the probabilistic switching task, we were able to replicate group-level behavior seen in previous studies. At higher set sizes, learning improved more slowly, and plateaued at a lower level (Fig S4a); asymptotic learning phase performance dropped in higher set sizes, while testing phase performance was impacted at lower set sizes (Fig S4b). Indeed, the effect of set size was statistically significant but of opposite sign in the two phases, replicating Collins, 2018.

While there is a slight difference in asymptotic learning phase performance in set size six between the two impulsivity groups, this effect does not survive more rigorous statistical tests: there was no significant correlation of impulsivity score and predictor weights (Fig S4c). Computational modeling results also did not illuminate any effects of impulsivity on underlying cognitive components captured by parameters of the best-fitting model (Fig S4d).

**Supplementary Figure Legend:**

Table S1.

| Scale | Behavioral Measure | Type | Coefficient | p–value |
| --- | --- | --- | --- | --- |
| Total impulsivity | Overall performance | Pearson | .089 | .46 |
|  | P(stay \| *r_t-1_=0*) | Spearman | .086 | .48 |
|  | P(stay \| ---) | Pearson | –.34 | .0046 |
|  | P(a\|ab) | Pearson | .30 | .015 |
| Positive Urgency | Overall performance | Spearman | –.0020 | .95 |
|  | P(stay \| *r_t-1_=0*) | Spearman | .19 | .10 |
|  | P(stay \| ---) | Spearman | –0.24 | .045 |
|  | P(a\|ab) | Spearman | .20 | .10 |
| Negative Urgency | Overall performance | Pearson | –.068 | .57 |
|  | P(stay \| *r_t-1_=0*) | Spearman | .21 | .077 |
|  | P(stay \| ---) | Pearson | –.20 | .09 |
|  | P(a\|ab) | Pearson | .21 | .082 |
| Sensation Seeking | Overall performance | Pearson | .12 | .32 |
|  | P(stay *r_t-1_=0*) | Spearman | .055 | .65 |
|  | P(stay \| ---) | Pearson | –.24 | .049 |
|  | P(a\|ab) | Pearson | .29 | .018 |
| Lack of Perseverance | Overall performance | Spearman | .25 | .035 |
|  | P(stay \| *r_t-1_=0*) | Spearman | –.21 | .079 |
|  | P(stay \| ---) | Spearman | –.11 | .38 |
|  | P(a\|ab) | Spearman | –.049 | .69 |
| Lack of Premeditation | Overall performance | Spearman | .047 | .70 |
|  | P(stay \| *r_t-1_=0*) | Spearman | –.078 | .52 |
|  | P(stay \| ---) | Spearman | –.17 | .15 |
|  | P(a\|ab) | Spearman | .021 | .87 |

Table S1. Table of correlation coefficients (and their corresponding p values). Correlations of total impulsivity score and subscale scores, and key behavioral measures (a priori: overall performance, *P(stay | r_t-1_=0)*; a posteriori: *P(stay | ---)*, *P(a|ab)*). Significant correlations with corrected p < 0.05 are highlighted in green, and uncorrected p < 0.05 highlighted in yellow (see methods). The subscale correlations are exploratory, and are thus uncorrected for multiple comparison.

Table S2.

| Scale | Predictors | Regressor weight | SE | z | p |
| --- | --- | --- | --- | --- | --- |
| Total impulsivity | Intercept | –0.10 | 0.03 | –3.08 | 0.002 |
|  | Score | 0.00 | 0.03 | 0.09 | 0.931 |
|  | pos_1 | 2.27 | 0.04 | 54.44 | 0.000 |
|  | neg_1 | –0.35 | 0.02 | –14.16 | 0.000 |
|  | pos_2 | 0.65 | 0.04 | 17.19 | 0.000 |
|  | neg_2 | –0.16 | 0.03 | –5.54 | 0.000 |
|  | pos_3 | 0.21 | 0.03 | 6.36 | 0.000 |
|  | neg_3 | 0.10 | 0.03 | 3.42 | 0.001 |
|  | score x pos_1 | 0.02 | 0.04 | 0.44 | 0.660 |
|  | score x neg_1 | –0.10 | 0.03 | –3.90 | 0.000 |
|  | score x pos_2 | 0.04 | 0.04 | 1.03 | 0.304 |
|  | score x neg_2 | 0.00 | 0.03 | 0.02 | 0.985 |
|  | score x pos_3 | 0.02 | 0.04 | 0.70 | 0.486 |
|  | score x neg_3 | 0.02 | 0.03 | 0.82 | 0.414 |
| Positive Urgency | Intercept | –0.10 | 0.033 | –3.10 | 0.002 |
|  | Score | 0.02 | 0.033 | 0.66 | 0.512 |
|  | pos_1 | 2.28 | 0.042 | 54.45 | 0.000 |
|  | neg_1 | –0.34 | 0.024 | –13.94 | 0.000 |
|  | pos_2 | 0.65 | 0.038 | 17.09 | 0.000 |
|  | neg_2 | –0.16 | 0.028 | –5.63 | 0.000 |
|  | pos_3 | 0.21 | 0.034 | 6.26 | 0.000 |
|  | neg_3 | 0.10 | 0.028 | 3.53 | 0.000 |
|  | score x pos_1 | –0.06 | 0.041 | –1.50 | 0.133 |
|  | score x neg_1 | –0.12 | 0.025 | –4.99 | 0.000 |
|  | score x pos_2 | –0.01 | 0.039 | –0.28 | 0.780 |
|  | score x neg_2 | 0.06 | 0.029 | 2.01 | 0.045 |
|  | score x pos_3 | 0.00 | 0.034 | –0.06 | 0.954 |
|  | score x neg_3 | 0.04 | 0.028 | 1.60 | 0.110 |
| Negative Urgency | Intercept | –0.10 | 0.033 | –3.12 | 0.002 |
|  | Score | 0.03 | 0.033 | 0.97 | 0.333 |
|  | pos_1 | 2.28 | 0.042 | 54.40 | 0.000 |
|  | neg_1 | –0.34 | 0.024 | –14.05 | 0.000 |
|  | pos_2 | 0.65 | 0.038 | 17.24 | 0.000 |
|  | neg_2 | –0.15 | 0.028 | –5.39 | 0.000 |
|  | pos_3 | 0.21 | 0.034 | 6.09 | 0.000 |
|  | neg_3 | 0.09 | 0.028 | 3.28 | 0.001 |
|  | score x pos_1 | –0.08 | 0.042 | –1.80 | 0.072 |
|  | score x neg_1 | –0.07 | 0.025 | –2.87 | 0.004 |
|  | score x pos_2 | –0.11 | 0.039 | –2.87 | 0.004 |
|  | score x neg_2 | –0.01 | 0.029 | –0.50 | 0.620 |
|  | score x pos_3 | 0.05 | 0.034 | 1.42 | 0.155 |
|  | score x neg_3 | 0.05 | 0.028 | 1.91 | 0.057 |
| Sensation Seeking | Intercept | –0.10 | 0.033 | –3.13 | 0.002 |
|  | Score | –0.01 | 0.033 | –0.41 | 0.678 |
|  | pos_1 | 2.28 | 0.042 | 54.25 | 0.000 |
|  | neg_1 | –0.35 | 0.024 | –14.17 | 0.000 |
|  | pos_2 | 0.66 | 0.038 | 17.31 | 0.000 |
|  | neg_2 | –0.15 | 0.028 | –5.38 | 0.000 |
|  | pos_3 | 0.21 | 0.034 | 6.21 | 0.000 |
|  | neg_3 | 0.10 | 0.028 | 3.45 | 0.001 |
|  | score x pos_1 | 0.13 | 0.042 | 3.20 | 0.001 |
|  | score x neg_1 | –0.10 | 0.024 | –4.17 | 0.000 |
|  | score x pos_2 | 0.10 | 0.038 | 2.73 | 0.006 |
|  | score x neg_2 | 0.07 | 0.028 | 2.67 | 0.008 |
|  | score x pos_3 | –0.03 | 0.033 | –1.04 | 0.297 |
|  | score x neg_3 | –0.01 | 0.028 | –0.52 | 0.601 |
| Lack of Perseverance | Intercept | –0.11 | 0.032 | –3.28 | 0.001 |
|  | Score | –0.05 | 0.033 | –1.49 | 0.136 |
|  | pos_1 | 2.29 | 0.042 | 54.21 | 0.000 |
|  | neg_1 | –0.34 | 0.024 | –14.07 | 0.000 |
|  | pos_2 | 0.65 | 0.038 | 17.13 | 0.000 |
|  | neg_2 | –0.16 | 0.028 | –5.66 | 0.000 |
|  | pos_3 | 0.22 | 0.034 | 6.35 | 0.000 |
|  | neg_3 | 0.09 | 0.028 | 3.37 | 0.001 |
|  | score x pos_1 | 0.10 | 0.043 | 2.27 | 0.023 |
|  | score x neg_1 | 0.05 | 0.024 | 2.00 | 0.046 |
|  | score x pos_2 | 0.11 | 0.038 | 2.84 | 0.005 |
|  | score x neg_2 | –0.07 | 0.028 | –2.61 | 0.009 |
|  | score x pos_3 | 0.07 | 0.034 | 2.07 | 0.039 |
|  | score x neg_3 | –0.03 | 0.028 | –0.90 | 0.370 |
| Lack of Premeditation | Intercept | –0.10 | 0.033 | –3.09 | 0.002 |
|  | Score | 0.01 | 0.033 | 0.18 | 0.856 |
|  | pos_1 | 2.28 | 0.042 | 54.51 | 0.000 |
|  | neg_1 | –0.34 | 0.024 | –14.09 | 0.000 |
|  | pos_2 | 0.65 | 0.038 | 17.13 | 0.000 |
|  | neg_2 | –0.15 | 0.028 | –5.48 | 0.000 |
|  | pos_3 | 0.21 | 0.034 | 6.34 | 0.000 |
|  | neg_3 | 0.10 | 0.028 | 3.40 | 0.001 |
|  | score x pos_1 | 0.00 | 0.042 | –0.07 | 0.941 |
|  | score x neg_1 | 0.00 | 0.025 | 0.08 | 0.940 |
|  | score x pos_2 | 0.05 | 0.039 | 1.26 | 0.207 |
|  | score x neg_2 | –0.09 | 0.029 | –2.96 | 0.003 |
|  | score x pos_3 | 0.02 | 0.035 | 0.49 | 0.624 |
|  | score x neg_3 | 0.00 | 0.028 | –0.01 | 0.992 |

Table S2. Table of coefficient values and p-values of regressor weights of GLME predicting an action based on reward outcomes from up to 3 trials past (t-3) and accounting for impulsivity (or subscale) and its interactions with reward outcomes. Significant correlations with corrected p < 0.05 are highlighted in green, and uncorrected p < 0.05 highlighted in yellow (see methods). Subscale statistics are part of exploratory analyses, and are thus uncorrected for multiple comparison.

Table S3.

| Scale | Model | Parameter | Spearman ρ | p–value |
| --- | --- | --- | --- | --- |
| Total impulsivity | RL | α | .017 | .89 |
|  |  | α_–_ | –.092 | .45 |
|  |  | st | –.11 | .35 |
|  |  | β | .025 | .84 |
|  | BI | p_reward_ | –.073 | .55 |
|  |  | p_switch_ | –.054 | .66 |
|  |  | st | –.13 | .28 |
|  |  | β | –.0001 | 1.0 |
| Positive Urgency | RL | α | .047 | .70 |
|  |  | α_–_ | –.065 | .59 |
|  |  | st | –.26 | .030 |
|  |  | β | –.10 | .39 |
|  | BI | p_reward_ | –.043 | .72 |
|  |  | p_switch_ | –.013 | .92 |
|  |  | st | –.23 | .052 |
|  |  | β | –.15 | .21 |
| Negative Urgency | RL | α | –.12 | .32 |
|  |  | α_–_ | .034 | .78 |
|  |  | st | –.21 | .076 |
|  |  | β | –.12 | .34 |
|  | BI | p_reward_ | .075 | .54 |
|  |  | p_switch_ | .042 | .73 |
|  |  | st | –.17 | .16 |
|  |  | β | –.14 | .24 |
| Sensation Seeking | RL | α | .03 | .81 |
|  |  | α_–_ | –.027 | .83 |
|  |  | st | –.044 | .72 |
|  |  | β | .054 | .66 |
|  | BI | p_reward_ | –.016 | .89 |
|  |  | p_switch_ | –.062 | .61 |
|  |  | st | –.084 | .49 |
|  |  | β | .076 | .53 |
| Lack of Perseverance | RL | α | .078 | .52 |
|  |  | α_–_ | –.27 | .026 |
|  |  | st | .17 | .17 |
|  |  | β | .24 | 0.048 |
|  | BI | p_reward_ | –.22 | .063 |
|  |  | p_switch_ | –.19 | .12 |
|  |  | st | .084 | .49 |
|  |  | β | .18 | .14 |
| Lack of Premeditation | RL | α | –.028 | .82 |
|  |  | α_–_ | –.1 | .39 |
|  |  | st | .043 | .72 |
|  |  | β | .038 | .75 |
|  | BI | p_reward_ | –.12 | .32 |
|  |  | p_switch_ | –.011 | .93 |
|  |  | st | .04 | .74 |
|  |  | β | .047 | .70 |

Table S3. Table of Spearman ρ coefficients and corresponding p-values from correlations of total impulsivity score and subscale scores and fit parameter values of the RL model (top) and BI model (bottom). For main analyses, correlations of fit parameter values and overall impulsivity were not significant. Significant correlations with corrected p < 0.05 are highlighted in green, and uncorrected p < 0.05 highlighted in yellow. Significant correlations for exploratory analyses of subscale scores are uncorrected for multiple comparison.


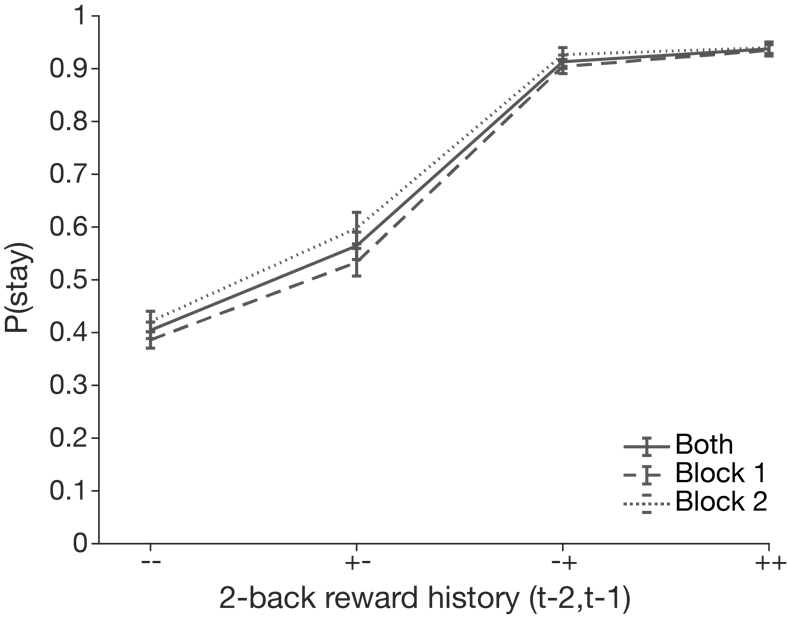


Figure S1. Probability of staying (*P(stay)*) conditioned by 2 trial-back reward history. Joint block behavior (solid line) is comparable to behavior from only block 1 (dashed line) and only block 2 (dotted line). Data points are averaged over all participants, bars represent standard error of the mean.


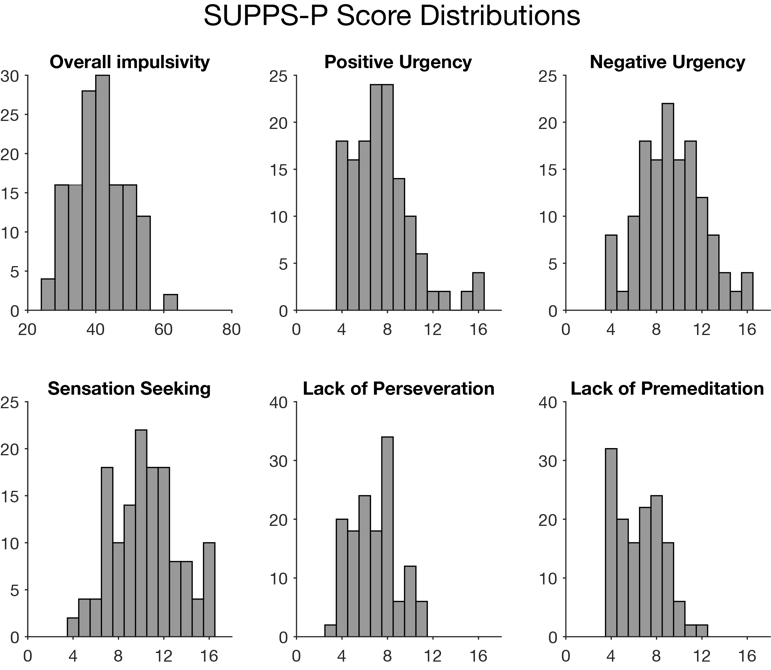


Figure S2. Histograms of total impulsivity score and subscale scores from S-UPPS-P. Normality of each distribution was established via Shapiro-Wilks test, α = 0.05. (A) Total impulsivity score (W = 0.981, p > 0.05). (B) Positive Urgency (W = 0.909, p < 0.05). (C) Negative Urgency (W = 0.978, p > 0.05). (D) Sensation Seeking (W = 0.973, p > 0.05). (E) Lack of Perseveration (W = 0.949, p < 0.05). (F) Lack of premeditation (W = 0.924, p < 0.05).


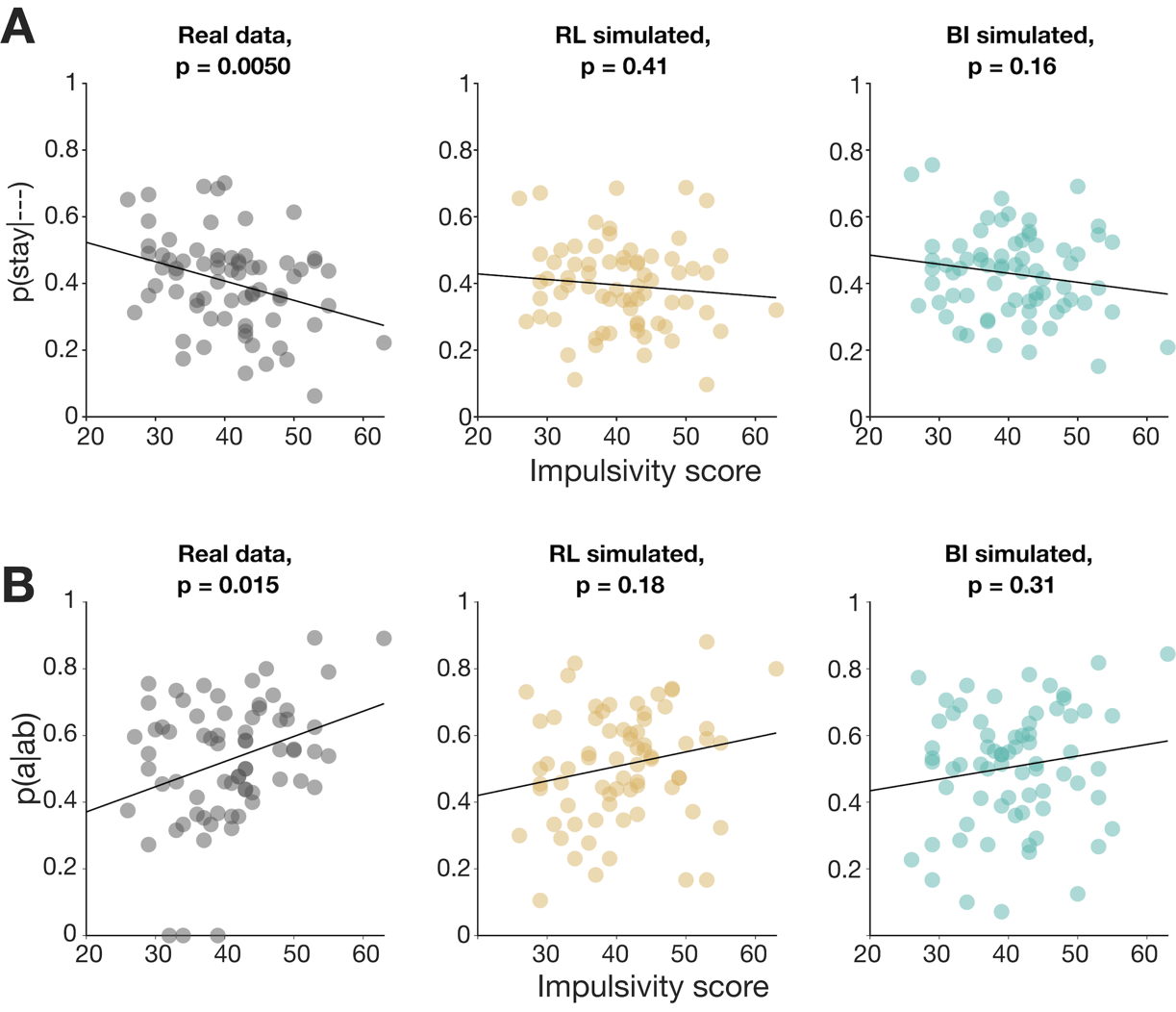


Figure S3. RL (brown) and BI (teal) models do not replicate relationship between impulsivity and *a posteriori* reward history-dependent behavior seen in real participant data (black). (A) Total impulsivity score vs. probability of staying after 3 consecutive unrewarded outcomes (p(stay|---)) of real participants, RL-simulated data, and BI-simulated data. (B) Total impulsivity score vs. returning to a previously unrewarded action following an unrewarded switch (p(a|ab)) of real participants, RL-simulated data, and BI-simulated data.


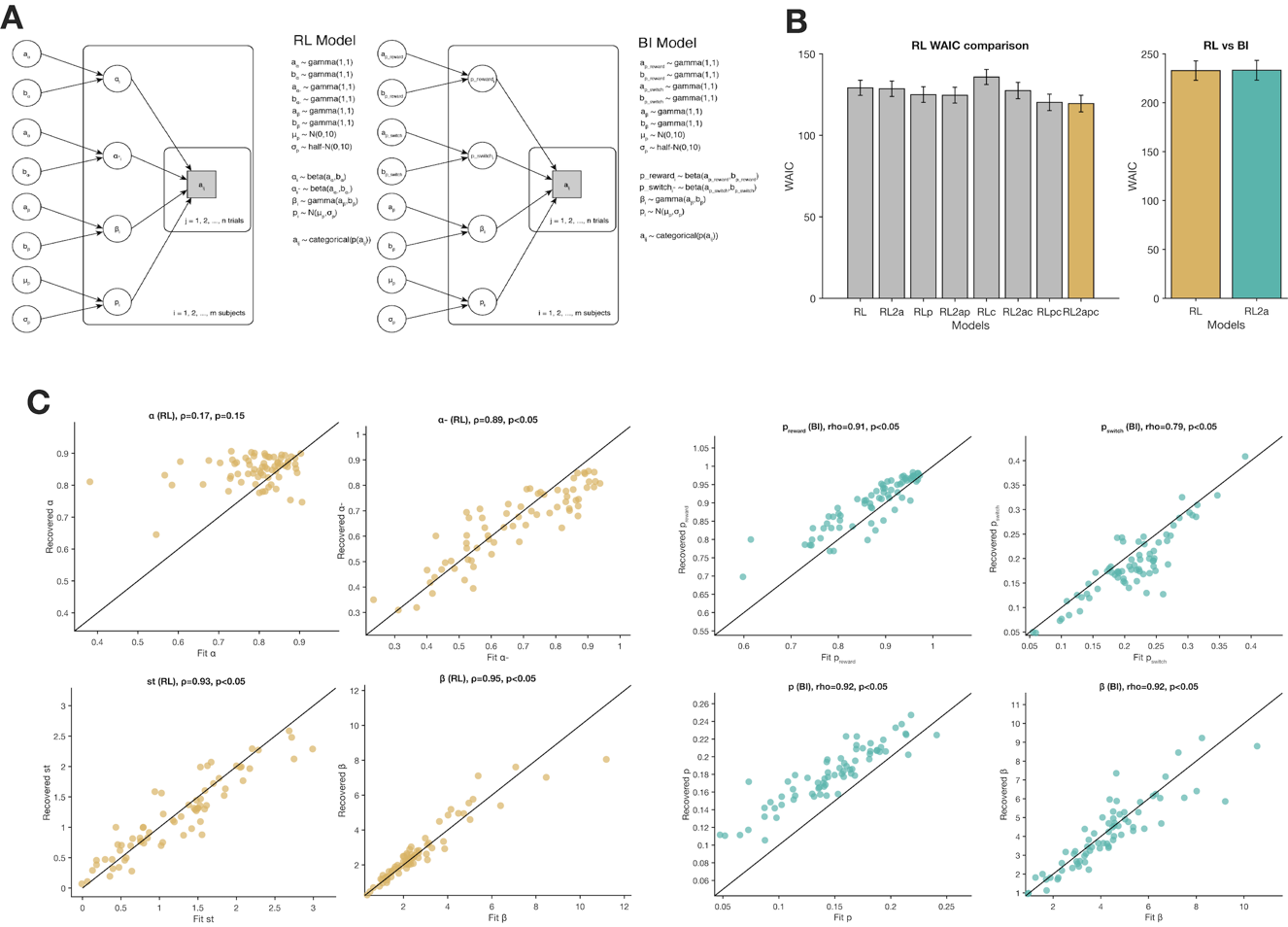


Figure S4. Computational modeling. (A) Graphical models of best-fitting Reinforcement Learning (RL) model (left) and Bayesian Inference (BI) model under the Bayesian hierarchical modeling framework. (B) WAIC score comparison. Left: comparison across RL nested model family (left, fitted over block 1 only); the best-fitting RL model (brown) has four free parameters and captures feedback-dependent learning rates, sticky choice behavior, and counterfactual updating (left). Right: comparison of the best-fitting RL model and the BI model; both were fit over both blocks of data jointly. (C) Generate and recover results of best-fitting RL model (left, brown), parameters from top-left, clockwise: *α*, *α_-_*, *st*, *β*; and BI model (right, teal), parameters from top-left, clockwise: *p_reward_*, *p_switch_*, *st*, *β*.


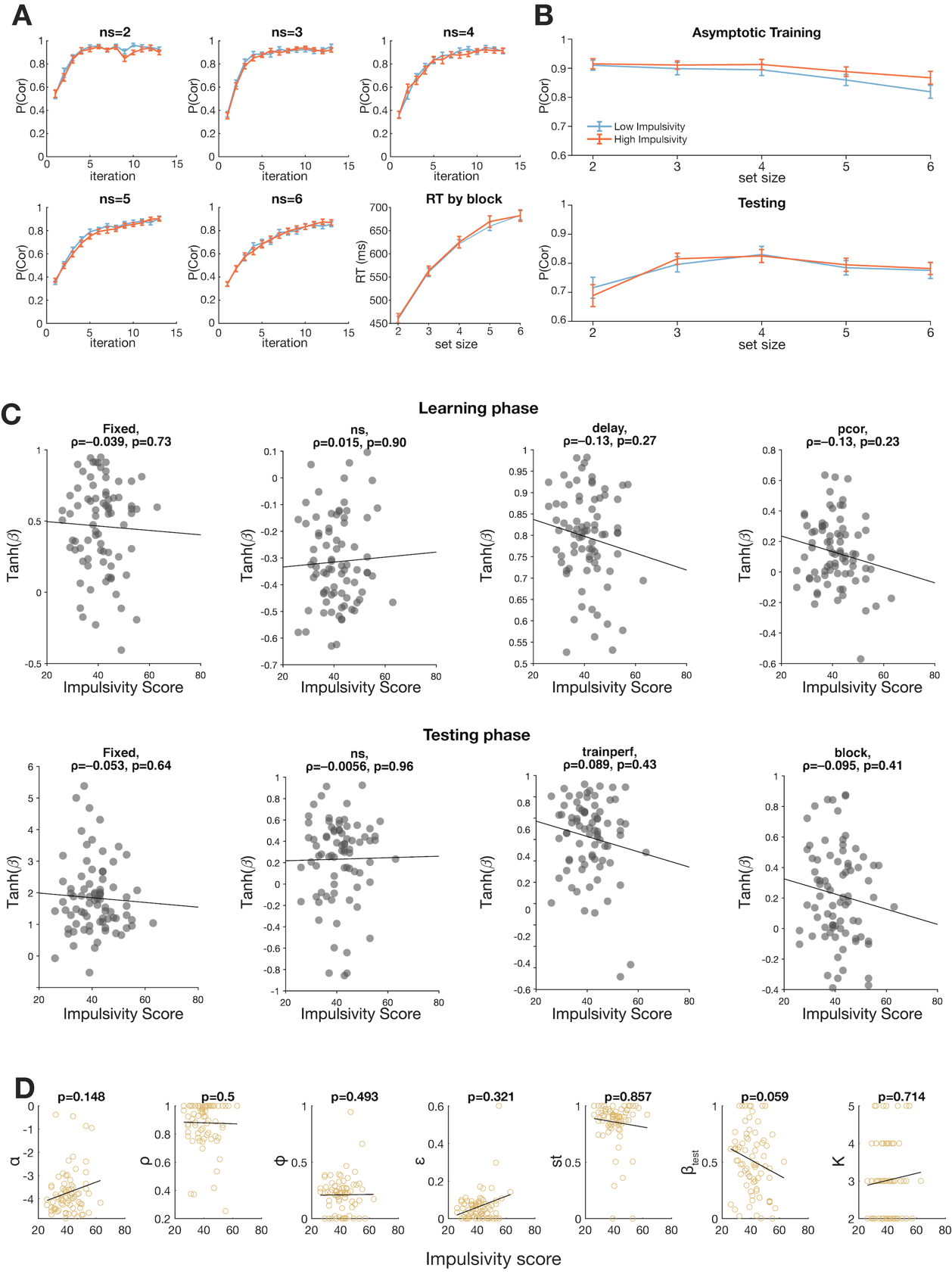


Figure S5. RLWM task. Median split for visualizing impulsivity effect (low impulsivity group = blue, high impulsivity group = orange). (A) Learning curves across set sizes, and average trial reaction time (ms) across set sizes. (B) Comparison of asymptotic learning phase behavior (top) and testing phase behavior (bottom) by set-size. (C) Impulsivity score is not significant related to the values of regressor weights predicting performance in the learning phase (left) and the testing phase (right). (D) Impulsivity score vs. fit parameter values of the RLWM model (Spearman correlations, all ps > 0.05).
